# Characterization of New *Ent*-kaurane Diterpenoids of Yunnan Arabica Coffee Beans

**DOI:** 10.1007/s13659-016-0099-1

**Published:** 2016-05-10

**Authors:** Rui Chu, Luo-Sheng Wan, Xing-Rong Peng, Mu-Yuan Yu, Zhi-Run Zhang, Lin Zhou, Zhong-Rong Li, Ming-Hua Qiu

**Affiliations:** State Key Laboratory of Phytochemistry and Plant Resources in West China, Kunming Institute of Botany, Chinese Academy of Sciences, Kunming, 650201 China; University of Chinese Academy of Sciences, Beijing, 100049 China

**Keywords:** *Coffea arabica* L., Green coffee beans, Diterpenoids, Structural elucidation

## Abstract

**Abstract:**

Five new *ent*-kaurane diterpenoids, named mascaroside III–V (**1**–**3**), and 20-nor-cofaryloside I–II (**4**–**5**), together with seven known diterpenoids, were isolated from methanol extracts of the green coffee beans of Yunnan Arabica Coffee. Their chemical structures were elucidated by extensive spectroscopic analyses. Meanwhile, cytotoxicity assay against HL-60, A-549, SMMC-7721, MCF-7 and SW480 cell lines showed that they have not evident inhibition of cytotoxicity.

**Graphical Abstract:**

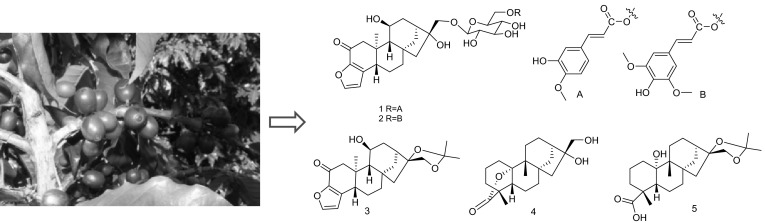

**Electronic supplementary material:**

The online version of this article (doi:10.1007/s13659-016-0099-1) contains supplementary material, which is available to authorized users.

## Introduction

**Fig. 1:**
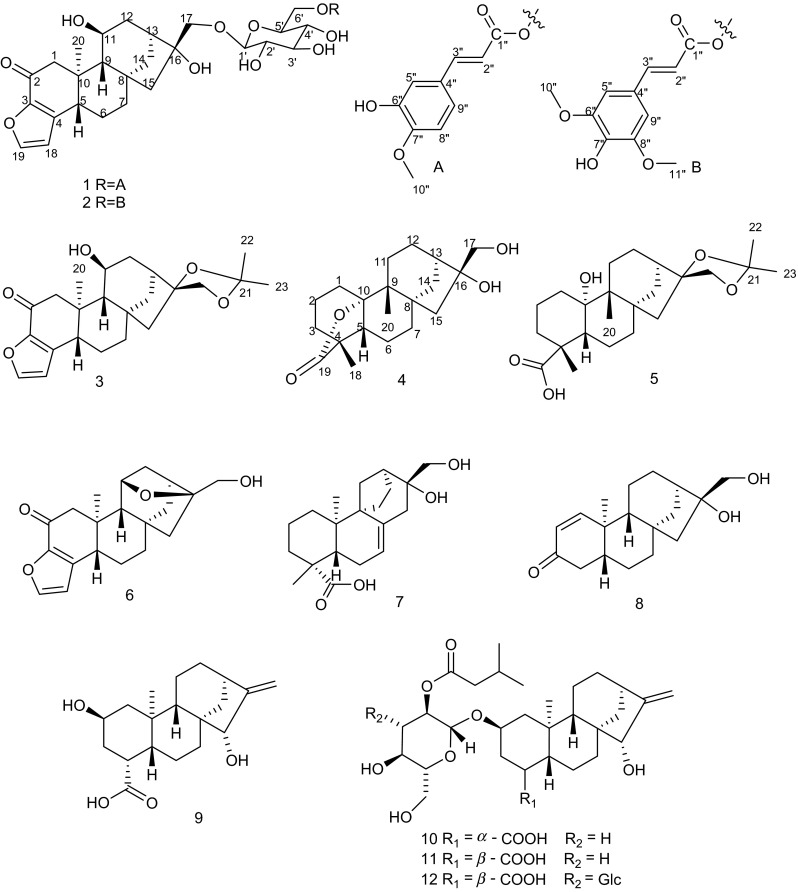
Structures of compounds **1**–**12**

*Coffea arabica* L., commonly known as coffee and widely distributed throughout the world, involving Africa, Latin America and Asia, is a very popular hot drink around the world because of its attractive aroma and unique taste [1, 2]. Previous studies have shown that coffee beans are consisted of caffeine, chlorogenic acids, saccharides [3–5], as well as diterpenoids, although, taking a minor proportion in the chemical constituents of coffee. However, due to their broad spectrum of biological activities, such as cytotoxicity, antioxidant, anti-inflammatory, researchers have carried out work on diterpenoids components from coffee beans, and found nearly 90 diterpenoids [6–9]. Yunnan Arabica Coffee was the *Coffea arabica* which were planted in Yunnan province. Along with the planting scale expanded to 120 thousand hectares, Yunnan province became a well-known cultivation base of *C. arabica* in the world. In order to investigate the chemical constituents of Yunnan Arabica Coffee and find bioactivity diterpenoids, we took Yunnan Arabica Coffee green beans collected in Dehong as the subject, and discovered five new *ent*-kaurane diterpenoids, along with seven known diterpenoids (Fig. 1). Herein, the isolation, structural elucidation, and their relevant bioactivities were also described.

## Results and Discussion

**Fig. 2:**
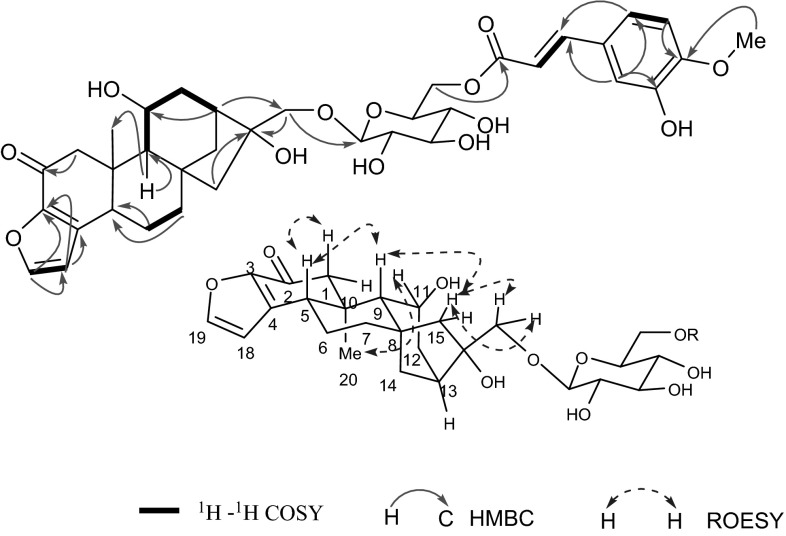
Key correlations in 2D NMR spectra of compound **1**

Mascaroside III (**1**) was isolated as a white amorphous powder. The molecular formula C_36_H_44_O_13_ was deduced from the molecular ion peak at m/z [M + Na]^+^ 707.2670 (calcd for C_36_H_44_O_13_Na, 707.2674) in HREIMS. The IR spectrum indicated that **1** possessed hydroxyl (3440 cm^−1^) and *α,β*-unsaturated ketone (1650 cm^−1^) groups. The ^13^C NMR (DEPT) data (Tables 2, 3) showed 36 carbon resonances, attributed to a monosaccharide, a cinnamic acids group and an aglycone moiety, and the aglycone moiety were classified as one methyl (*δ*_C_ 15.7), 7 methylenes (including one oxygenated), 6 methines (including one oxygenated, two olefinic), and 6 quaternary carbons (including one oxygenated, two olefinic, and one carbonyl). These data (Tables 1, 2, 3) were similar to those of mascaroside I [10, 11] except for 10 additional signals for a cinnamic acids group. The coupling constant (*J*_2″, 3″_ = 15.9 Hz) suggested the double bond of the cinnamic acid group was *trans*. Besides, *δ*_H_ 6.80(d, *J* = 8.2 Hz), 7.10(d, *J* = 8.2 Hz) were due to ortho-aromatic hydrogen suggested the two oxygenated *sp*^2^ quaternary carbons were at C-6″ and C-7″, along with CH_3_-10″ linked to OH-7″ confirmed by the HMBC correlations from H_3_-10″ (*δ*_H_ 3.88) to C-7″. Further, the OH-6′ in the glucose and COOH-1″ in *tran*s-cinnamic acids group formed into ester were confirmed by HMBC correlations of H-6′ (*δ*_H_ 4.29, 4.69) to C-1″ (*δ*_C_ 169.5). The relative configuration of glucose anomeric proton was confirmed as *β* on the basis of the coupling constant (*J*_1′, 2′_ = 7.8 Hz). Furthermore, the glucose was identified as D-form by GC analysis comparing with a standard after acid hydrolysis [12, 13]. The relative configuration of **1** was same with mascaroside I by comparison of the NMR data. Furthermore, owing to the greatly predominant occurrence of this enantiomeric form in nature world, and until now, all the kaurene skeleton diterpenoids have been isolated from *Coffea arabica* were *ent*-kaurene series. Therefore, compound **1** was confirmed as an *ent*-kauranoid with the negative specific rotation value (−126.67) confirmed it further. *ent*-Kauranoid with the configuration of C-20 being *α*-orientated and H-5, H-9 being *β*-orientated. The key ROESY correlations of H-11 (*δ*_H_ 3.86)/H-20 (*δ*_H_ 0.78), and H-5 (*δ*_H_ 2.83)/H-9 (*δ*_H_ 1.61), H-9/H-15b (*δ*_H_ 2.12), and H-15b/H_2_-17 (*δ*_H_ 3.59, 4.71) allowed to assign H-11 as *α*-orientated, and CH_2_OH-17 as *β*-orientated, separately [14]. Hence, the structure of **1** was determined and named as mascaroside III (Fig. 2).

**Table 1:** ^1^H NMR spectral data of compounds **1**–**5** [*δ* in ppm, *J* in Hz]

Position	**1** ^a^	**2** ^a^	**3** ^b^	**4** ^a^	**5** ^b^
1	2.39 (d, 16.3)	2.42 (d, 16.3)	2.33 (d, 16.1)	1.28 (dd, 13.6, 4.4)	0.94 (m)
	2.74 (d, 16.3)	2.77 (d, 16.3)	2.83 (d, 16.1)	1.81 (m)	2.01 (dd, 14.8, 5.9)
2	^–^	^–^	^–^	1.19 (m)	1.52 (m)
	^–^	^–^	^–^	1.72 (m)	1.64 (m)
3	^–^	^–^	^–^	1.58 (m)	1.01 (m)
	^–^	^–^	^–^	1.65 (m)	2.10 (m)
5	2.83 (br d, 14.2)	2.85 (br d, 12.6)	2.77 (dd, 12.4, 2.4)	1.94 (dd, 14.0, 4.8)	1.53 (m)
6	1.61 (m)	1.63 (m)	1.62 (m)	1.57 (m)	1.84 (m)
	2.01 (m)	2.03 (m)	1.99 (m)	1.86 (m)	1.89 (m)
7	1.72 (m)	1.73 (m)	1.78 (m, 2H)	1.77 (m)	1.64 (m)
	2.04 (m)	2.06 (m)		1.90 (m)	2.22 (d, 12.1)
9	1.61 (m)	1.63 (m)	1.61 (m)	^–^	^–^
11	3.86 (m)	3.89 (m)	3.93 (m)	1.72 (m)	1.44 (m)
	^–^	^–^	^–^	2.20 (dd, 13.8, 5.8)	1.75 (m)
12	1.72 (m)	1.73 (m)	1.81 (m)	1.61 (m)	1.45 (m)
	1.78 (m)	1.78 (m)	1.95 (m)	1.63 (m)	1.51 (m)
13	2.10 (m)	2.13 (m)	2.15 (m)	1.90 (m)	2.11 (m)
14	1.74 (m)	1.73 (m)	1.57 (m)	1.77 (m)	1.50 (m)
	1.77 (m)	1.76 (m)	1.85 (m)	1.91 (m)	1.65 (m)
15	1.37 (d, 14.3)	1.40 (d, 14.4)	1.85 (m)	1.39 (d, 14.7)	1.53 (m)
	2.12 (m)	2.15 (d, 14.4)	2.40 (m)	2.02 (d, 14.7)	2.44 (d, 15.0)
17	3.59 (d, 10.2)	3.53 (d, 10.2)	4.10 (d, 9.3)	3.64 (d, 11.5)	3.89 (d, 8.6)
	4.71 (d, 10.2)	4.74 (d, 10.2)	4.27 (d, 9.3)	3.70 (d, 11.5)	4.04 (d, 8.6)
18	6.58 (s)	6.61 (s)	6.42 (d, 1.4)	1.06 (s)	1.23 (s)
19	7.78 (s)	7.81 (s)	7.60 (d, 1.4)	^–^	^–^
20	0.78 (s)	0.81 (s)	0.87 (s)	1.16 (s)	1.08 (s)
22	^–^	^–^	1.37 (s)	^–^	1.39 (s)
23	^–^	^–^	1.36 (s)	^–^	1.36 (s)

^a^Data were measured at 600 MHz in CD_3_OD

^b^Data were measured at 600 MHz in CDCl_3_

**Table 2:** ^13^C NMR spectral data of compounds **1**–**5** [*δ* in ppm]

Position	**1** ^a^	**2** ^a^	**3** ^b^	**4** ^a^	**5** ^b^
1	54.3 (t)	54.3 (t)	53.4 (t)	31.8 (t)	29.7 (t)
2	187.7 (s)	187.6 (s)	185.2 (s)	19.8 (t)	28.1 (t)
3	148.0 (s)	147.9 (s)	146.6 (s)	35.8 (t)	37.6 (t)
4	144.8 (s)	144.8 (s)	142.1 (s)	50.1 (s)	49.5 (s)
5	45.8 (d)	45.8 (d)	44.8 (d)	52.2 (d)	50.1 (d)
6	23.4 (t)	23.3 (t)	22.4 (t)	21.6 (t)	21.8 (t)
7	37.1 (t)	37.0 (t)	39.6 (t)	41.4 (t)	39.3 (t)
8	45.2 (s)	45.2 (s)	42.3 (s)	43.5 (s)	43.8 (s)
9	62.1 (d)	62.1 (d)	61.0 (d)	45.9 (s)	43.8 (s)
10	43.8 (s)	43.8 (s)	43.9 (s)	90.6 (s)	77.1 (s)
11	66.1 (d)	66.1 (d)	65.2 (d)	34.4 (t)	36.2 (t)
12	37.5 (t)	37.5 (t)	36.7 (t)	24.9 (t)	19.0 (t)
13	46.5 (d)	46.5(d)	44.7 (d)	44.8 (d)	44.2 (d)
14	41.3 (t)	41.2 (t)	38.0 (t)	33.5 (t)	32.3 (t)
15	52.0 (t)	52.0 (t)	55.1 (t)	50.9 (t)	50.2 (t)
16	82.4 (s)	82.3 (s)	89.3 (s)	84.9 (s)	89.2 (s)
17	75.4 (t)	75.4 (t)	70.5 (t)	66.2 (t)	69.7 (t)
18	111.4 (d)	111.4 (d)	110.0 (d)	17.5 (q)	29.1 (q)
19	150.4 (d)	150.4 (d)	148.2 (d)	183.0 (s)	183.2 (s)
20	15.7 (q)	15.7 (q)	15.3 (q)	19.3 (q)	17.5 (q)
21	^–^	^–^	108.1 (s)	^–^	104.2 (s)
22	^–^	^–^	26.8 (q)	^–^	26.9 (q)
23	^–^	^–^	26.6 (q)	^–^	26.8 (q)

^a^Data were measured at 150 MHz in CD_3_OD

^b^Data were measured at 150 MHz in CDCl_3_

**Table 3:** ^1^H NMR and ^13^C NMR of the glucose and cinnamic acids group [*δ* in ppm, *J* in Hz]

	^1^H NMR		^13^C NMR	
	1	2	1	2
1′	4.32 (d, 7.8)	4.34 (d, 7.5)	104.8 (d)	104.8 (d)
2′	3.22 (m)	3.26 (t, 8.3)	75.4 (d)	75.4 (d)
3′	3.40 (m)	3.43 (m)	77.6 (d)	77.6 (d)
4′	3.40 (m)	3.43 (m)	71.5 (d)	71.5 (d)
5′	3.50 (m)	3.61 (d, 10.1)	75.5 (d)	75.5 (d)
6′	4.29 (m)	4.32 (d, 4.7)	63.9 (t)	63.8 (t)
	4.69 (m)	4.73 (m)	^–^	^–^
1″	^–^	^–^	169.5 (s)	169.5 (s)
2″	6.42 (d, 15.9)	6.49 (d, 15.9)	115.2 (d)	115.7 (d)
3″	7.65 (d, 15.9)	7.67 (d, 15.9)	147.4 (d)	147.6 (d)
4″	^–^	^–^	127.6 (s)	126.9 (s)
5″	7.20 (s)	6.95 (s)	111.8 (d)	107.0 (d)
6″	^–^	^–^	150.8 (s)	149.5 (s)
7″	^–^	^–^	149.4 (s)	139.8 (s)
8″	6.80 (d, 8.2)	^–^	116.5 (d)	149.5 (s)
9″	7.10 (d, 8.2)	6.95 (s)	124.3 (d)	107.0 (d)
10″	3.88 (s)	3.89 (s)	56.5 (q)	56.9 (q)
11″	^–^	3.89 (s)	^–^	56.9 (q)

*δ*_H_ Data were measured at 600 MHz in CD_3_OD

*δ*_C_ Data were measured at 150 MHz in CD_3_OD

Mascaroside IV (**2**) had the molecular formula of C_37_H_46_O_14_ according to the HRESIMS analysis at m/z [M + Na]^+^ 737.2786 (calcd for C_37_H_46_O_14_Na, 737.2780). The 1D NMR data (Tables 1, 2, 3) of **2** was identical to that of **1**, except that the cinnamic acids group in **2** was substituted by one more oxygenated methyl, which was further verified by the HMBC correlations from H-10″, and H-11″ (*δ*_H_ 3.89) to C-6″, and C-8″ (*δ*_C_ 149.5). The chiral centers of **2** were same with those of compound **1.** Therefore, the structure of **2** was elucidated as shown and given the name mascaroside IV.

Mascaroside V (**3**) was isolated as white powder. The HRESIMS of **3** showed an ion peak at m/z [M + Na]^+^ 409.1987 (calcd for 409.1985) suggesting a molecular formula C_23_H_30_O_5._ The 1D NMR spectroscopic features showed it similar to the aglycone moiety of **1** with the differences being that three more carbon atoms (*δ*_C_ 108.1 s, 26.8 q, 26.6 q). HSQC together with HMBC spectral signals, showed that CH_3_-22 (*δ*_C_ 26.8), CH_3_-23 (*δ*_C_ 26.6) were located at the same *sp*^3^ quaternary carbon (C-21 *δ*_C_ 108.1 s) which was confirmed by correlations from H-22, H-23 to C-21. This three carbons group was substituted at 16-OH, 17-OH to form a ketal ring [15] based on HMBC correlations from H_2_-17 (*δ*_H_ 4.10, 4.27) to C-21.The ROESY correlations were similar to compound **1**, consequently, the structure of **3** was confirmed as mascaroside V.

**Fig. 3:**
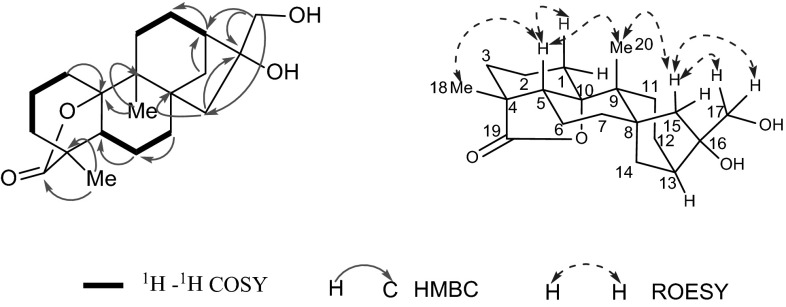
Key correlations in 2D NMR spectra of compound **4**

20-nor-cofaryloside I (**4**) a white amorphous powder, displayed a [M + Na]^+^ 357.2032 (calcd for 357.2036) in HREIMS, consistent with the molecular formula of C_20_H_30_O_4_, indicating 6 degrees of unsaturation. The compound **4** displayed similar characteristic signals to 10α,16α,17-trihydroxy-9α-methyl-15-oxo-20-nor-kauran-19-oic acid γ-lactone (**A**) [16, 17] except for the absence of the carbonyl groups at C-15. This was confirmed by the chemical shift at C-15 (*δ*_C_ 50.9) in **4** which was upfield shifted comparing to that in A (*δ*_C_ 224.0). Besides, the HMBC correlations from H_2_-15 (*δ*_H_ 1.39, 2.02) to C-16 (*δ*_C_ 84.9) and C8 (*δ*_C_ 43.5) also supported these change. On biogenetic grounds, compound **4** as an *ent*-kaurane with the configuration of H-5 and H-9 being *β*-orientated, while OH-10 being *α*-orientated. Its ROESY correlations showed cross peaks of H-5 (*δ*_H_ 1.94)/Me-18 (*δ*_H_ 1.06), H-5/Me-20 (*δ*_H_ 1.16), Me-20/H-15b (*δ*_H_ 2.02), H-15b/H_2_-17 (*δ*_H_ 3.64, 3.70), revealing that the orientation of C-18, C-20 and C-17 was *β*-orientated (Fig. 3). Thereupon, the structure of **4** was identified as 10*α*,16*α*,17-trihydroxy-9*β*-methyl-20-nor-*ent*-kauran-19-oic acid *γ*-lactone, and named 20-nor-cofaryloside I.

**Fig. 4:**
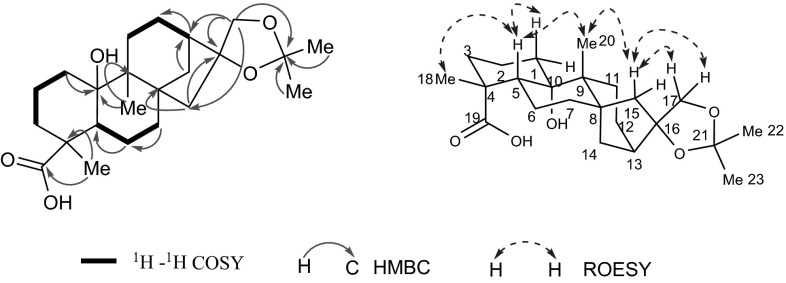
Key correlations in 2D NMR spectra of compound **5**

20-nor-cofaryloside II (**5**) possessed the molecular formula of C_23_H_36_O_5_, according to the HRESIMS analysis at m/z [M − H]^−^ 391.2488 (calcd for 391.2490)_._ Analyses of **5**′s ^1^H and ^13^C NMR data indicated the existence of 23 carbon resonances, and C-21 (*δ*_C_ 104.2 s), C-22 (*δ*_C_ 26.9 q) and C-23 (*δ*_C_ 26.9 q) suggesting that the structure of compound **5** had the same ketal ring as compound **3** and this deduction was further supported by HMBC correlations from H_2_-17, H_3_-22 and H_3_-23 to C-21. The other 20 carbon signals showed that compound 5 was similar to **4** while the chemical shift of the oxygenated *sp*^3^ quaternary carbon C-10 (*δ*_C_ 77.1) of **5** was upfield shifted comparing to that of **4** (*δ*_C_ 90.6), and the molecular weight of **5** was 18 units more than that of **4** along with the degrees of unsaturation decrease by one. Therefore, the lactone linkage which assigned between COOH-19 and OH-10 was ring opened in **5**. The relative configuration of **5** (Fig. 4) was same with **4**. Thus, the structure was defined as 10*α*-hydroxy- 16*α*,17-[(1-methylethylidene)bis(oxy)]-9*β*-methyl-20-nor-*ent*-kauran-19-oic acid, and named 20-nor- cofaryloside II.

Seven known diterpenoids were also obtained from this genus, bengalensol [18], villanovane [10], tricalysione A [14], 2*β*,16*α*,17-trihydroxy-*ent*-kauran-19*α*-oic acid [19], 2-O-(2-O-isovaleryl-*β*- d-gluco-pyranosyl)-4*α*-atractyligenin [20], 2-O-(2-O-isovaleryl-*β*-d-gluco-pyranosyl)-4*β*-atrac- tyligenin [20], 3-O-*β*-d-glucopyranosyl-2-O-(2-O-isovaleryl-*β*-d-gluco-pyranosyl)-4*β*-atracty ligenin [20]. Their structures were identified by comparison of their NMR data with literature data.

Compounds **1**–**5**, **7**, **8** were evaluated for cytotoxicity against HL-60, A-549, SMMC-7721, MCF-7 and SW480 cell lines. Unfortunately, they were inactive against all test cells (Electronic supplementary material, Table S1).

## Experimental Section

### General Experimental Procedures

1D and 2D NMR spectra were obtained on a Bruker Avance III 600 MHz spectrometer (Bruker Biospin GmbH, Karlsruhe, Germany). HREIMS was measured on Waters Xevo TQ-S and Waters Autospec Premier P776 mass spectrometers (Waters, Milford, MA, USA). HRESIMS were recorded on an Agilent 6200 Q-TOF MS system (Agilent Technologies, Santa Clara, CA, USA). UV spectra were recorded on a Shimadzu UV-2401PC (Shimadzu, Kyoto, Japan). Optical rotations were obtained on a JASCO P-1020 digital polarimeter (Horiba, Kyoto, Japan). IR spectra were detected on Bruker Tensor 27 FTIR (KBr pellets) spectrometers. Sephadex LH-20 (Amersham Biosciences, Upssala, Sweden) and silica gel (Qingdao Haiyang Chemical Co., Ltd) were used for column chromatography (CC). Preparative high performance liquid chromatography (prep-HPLC) was performed on an Agilent 1100 liquid chromatography system equipped with Zorbax SB-C18 columns (9.4 mm × 250 mm) and a DAD detector (Agilent Technologies, Santa Clara, CA, USA). Thin-layer chromatography was performed on precoated TLC plates (200–250 μm thickness, silica gel 60 F254, Qingdao Marine Chemical, Inc.), and spots were visualized by heating after spraying.

### Plant Material

The green coffee beans of *Coffea arabica* L. were harvested in December 2014 and identified by Hong-bo Zhang, Dehong Institute of Tropical Agriculture. A voucher specimen of *C*. *arabica* was deposited in the Herbarium of Kunming Institute of Botany, Chinese Academy of Sciences (No. KCF 201412).

### Extraction and Isolation

The air-dried and powdered green Dehong coffee beans (18 kg) were extracted with 95 % methanol three times, and then the combined filtrates were concentrated under reduced pressure to give a crude extract (5 kg). The crude extract was suspended in H_2_O and extracted with petroleum ether (PE), ethyl acetate (EtOAc), respectively. The EtOAc layer (160 g) was got rid of saccharides by D101 and then subjected to RP-18 column chromatography which eluted with MeOH–H_2_O (gradient from 15:85 to 100:0 v/v) to yield four fractions: fraction 1 (20 g), fraction 2 (19 g), fraction 3 (30 g), and fraction 4 (24 g). Fraction 4 was separated on silica gel CC using a CHCl_3_–MeOH gradient solvent system (80:0 → 1:1, v/v) to obtain eight subfractions (fraction 4–1 to 4–8). Then fraction 4–6 (6.5 g) was chromatographed on RP-18 CC (MeOH–H_2_O 10:90–100:0 v/v), Sephadex LH-20 (MeOH) and then purified by semipreparative HPLC (elute with CH_3_CN–H_2_O 15–75 %, 30 min) to afford **1** (5 mg), **2** (7 mg), **10** (2 mg), **11** (8 mg), **12** (40 mg). In the same way, **5** (14 mg), **6** (4 mg), **7** (17 mg) were isolated from fraction 4-2 (370 mg) and **3** (2 mg), **4** (5 mg), **8** (3 mg), **9** (6 mg) were isolated from fraction 4-3 (2.7 g).

### Mascaroside III (**1**)

White amorphous powder; $\left[ \alpha \right]_{\text{D}}^{19}$ −126.67 (*c* 0.150, MeOH); UV (MeOH) *λ*_max_ (log *ε*) 329 (4.87), 281 (4.14), 240 (3.94), 216 (3.97), 202 (4.05) nm; IR (KBr) *ν*_max_ 3439, 2923, 2878,1703, 1657, 1515, 1437, 1270, 1165, 1126, 1033 cm^−1^; ^1^H (600 MHz, CD_3_OD) and ^13^C NMR (150 MHz, CD_3_OD) data, Tables 1, 2 and 3; ESIMS *m/z* 707 [M + Na]^+^; HREIMS *m/z* [M + Na] ^+^ 707.2670 (calcd for C_36_H_44_O_13_Na, 707.2674).

### Mascaroside IV (**2**)

White amorphous powder; $\left[ \alpha \right]_{\text{D}}^{19}$ −107.20 (*c* 0.213, MeOH); UV (MeOH) *λ*_max_ (log *ε*) 332 (4.00), 279 (4.03), 241 (4.03), 202 (4.12) nm; IR (KBr) *ν*_max_ 3439, 2924, 2855, 1706, 1663, 1513, 1436, 1258, 1155, 1113, 1042 cm^−1^; ^1^H (600 MHz, CD_3_OD) and ^13^C NMR (150 MHz, CD_3_OD) data, Tables 1, 2, and 3; ESIMS *m/z* 737 [M + Na]^+^; HREIMS *m/z* [M + Na]^+^ 737.2786 (calcd for C_37_H_46_O_14_Na, 737.2780).

### Mascaroside V (**3**)

White amorphous powder; $\left[ \alpha \right]_{\text{D}}^{19}$ −132.81 (*c* 0.243, CHCl_3_); UV (CHCl_3_) *λ*_max_ (log *ε*) 276 (4.05), 232 (3.71), 208 (3.67), 198 (3.64) nm; IR (KBr) *ν*_max_ 3492, 2925, 2866, 1665, 1436, 1368, 1208, 1128, 1049 cm^−1^; ^1^H (600 MHz, CDCl_3_) and ^13^C NMR (150 MHz, CDCl_3_) data, Tables 1,2; ESIMS *m/z* 409 [M + Na]^+^; HREIMS *m/z* [M + Na]^+^ 409.1987 (calcd for C_23_H_30_O_5_Na, 409.1985).

### 20-Nor-cofaryloside I (**4**)

white amorphous powder; $\left[ \alpha \right]_{\text{D}}^{19}$ −7.77 (*c* 0.206, MeOH); UV (MeOH) *λ*_max_ (log *ε*) 274 (1.85), 206 (2.59) nm; IR (KBr) *ν*_max_ 3439, 2931, 2870, 1759, 1632, 1443, 1382, 1174, 1136, 1148, 935 cm^−1^; ^1^H (600 MHz, CD_3_OD) and ^13^C NMR (150 MHz, CD_3_OD) data, Tables 1,2; ESIMS *m/z* 737 [M + Na]^+^; HREIMS *m/z* [M + Na]^+^ 357.2032 (calcd for C_20_H_30_O_4_Na_,_ 357.2036).

### 20-Nor-cofaryloside II (**5**)

White amorphous powder; $\left[ \alpha \right]_{\text{D}}^{18}$ −51.01 (*c* 0.105, CHCl_3_); UV (CHCl_3_) *λ*_max_ (log *ε*) 280 (2.85), 241 (2.96), 229 (2.83), 192 (1.77) nm; IR (KBr) *ν*_max_ 3442, 2936, 2872, 1718, 1699, 1464, 1373, 1252, 1213, 1052, 970 cm^−1^; ^1^H NMR (600 MHz, CDCl_3_) and ^13^C NMR (150 MHz, CDCl_3_) data, Tables 1 and 2; ESIMS *m/z* 391 [M − H]^−^; HREIMS *m/z* [M – H]^−^ 391.2488 (calcd for C_23_H_35_O_5_, 391.2490)_._

### Cytotoxicity Assay

The cytotoxicity against HL-60, A-549, SMMC-7721, MCF-7 and SW480 cell lines of compounds **1**–**5**, **7**, **8** were tested by using MTS method. MTS [3-(4,5-dimethylthiazol-2-yl)-5(3-carboxymethoxyphenyl)-2-(4-sulfopheny)-2*H*-tetrazolium] is an analogue of MTT [21], which can be reduced into soluble formazan by succinate dehydrogenase in mitochondria of living cells. Moreover, the optical density value of formazan (490 nm) is proportional to the number of living cells.

## Electronic supplementary material

Below is the link to the electronic supplementary material.

Supplementary material 1 (DOCX 3746 kb)
