# Supplementary material for: Characterization of New Ent-kaurane Diterpenoids of Yunnan Arabica Coffee Beans
Source: Nat Prod Bioprospect. 2016 May 10;6(4):217–23. doi: 10.1007/s13659-016-0099-1 (PMC4940257; doi:10.1007/s13659-016-0099-1)

**Supplementary data**

**Characterization of New *ent*-kaurane Diterpenoids of Yunnan Arabica Coffee Beans**

**Rui Chu^a,b^, Luo-Sheng Wan^a^, Xing-Rong Peng^a^, Mu-Yuan Yu^a,b^, Zhi-Run Zhang^a^, Lin Zhou^a^, Zhong-Rong Li^a^, Ming-Hua Qiu^a,b*^**

a State Key Laboratory of Phytochemistry and Plant Resources in West China, Kunming Institute of Botany, Chinese Academy of Sciences, Kunming 650201, China

b University of Chinese Academy of Sciences, Beijing 100049, China

**Corresponding Authors**

*Ming-Hua Qiu

E-mail: [mhchiu@mail.kib.ac.cn](mailto:mhchiu@mail.kib.ac.cn)

Tel: 86-871-65223327, Fax: 86-871-65223255,

**Contents**

| Figures | Content |
| --- | --- |
| S1 | bioassay |
| S2 | ^1^H NMR spectrum of compound **1** (CD_3_OD). |
| S3 | ^13^C NMR and DEPT spectra of compound **1** (CD_3_OD). |
| S4 | HSQC spectrum of compound **1** (CD_3_OD ). |
| S5 | ^1^H-^1^H COSY spectrum of compound **1** (CD_3_OD ) |
| S6 | HMBC spectrum of compound **1** (CD_3_OD). |
| S7 | ROESY spectrum of compound **1** (CD_3_OD ). |
| S8 | HREIMS spectrum of compound **1** |
| S9 | IR spectrum of compound **1** |
| S10 | ^1^H NMR spectrum of compound **2** (CD_3_OD). |
| S11 | ^13^C NMR and DEPT spectra of compound **2** (CD_3_OD). |
| S12 | HSQC spectrum of compound **2** (CD_3_OD ). |
| S13 | ^1^H-^1^H COSY spectrum of compound **2** (CD_3_OD ) |
| S14 | HMBC spectrum of compound **2** (CD_3_OD). |
| S15 | ROESY spectrum of compound **2** (CD_3_OD ). |
| S16 | HREIMS spectrum of compound **2** |
| S17 | IR spectrum of compound **2** |
| S18 | ^1^H NMR spectrum of compound **3** (CDCl_3_). |
| S19 | ^13^C NMR and DEPT spectra of compound **3** (CDCl_3_). |
| S20 | HSQC spectrum of compound **3** (CDCl_3_). |
| S21 | ^1^H-^1^H COSY spectrum of compound **3** (CDCl_3_) |
| S22 | HMBC spectrum of compound **3** (CDCl_3_). |
| S23 | ROESY spectrum of compound **3** (CDCl_3_). |
| S24 | HREIMS spectrum of compound **3** |
| S25 | IR spectrum of compound **3** |
| S26 | ^1^H NMR spectrum of compound **4** (CD_3_OD). |
| S27 | ^13^C NMR and DEPT spectra of compound **4** (CD_3_OD). |
| S28 | HSQC spectrum of compound **4** (CD_3_OD ). |
| S29 | ^1^H-^1^H COSY spectrum of compound **4** (CD_3_OD ) |
| S30 | HMBC spectrum of compound **4** (CD_3_OD). |
| S31 | ROESY spectrum of compound **4** (CD_3_OD ). |
| S32 | HREIMS spectrum of compound **4** |
| S33 | IR spectrum of compound **4** |
| S34 | ^1^H NMR spectrum of compound **5** (CDCl_3_). |
| S35 | ^13^C NMR and DEPT spectra of compound **5** (CDCl_3_). |
| S36 | HSQC spectrum of compound **5** (CDCl_3_). |
| S37 | ^1^H-^1^H COSY spectrum of compound **5** (CDCl_3_) |
| S38 | HMBC spectrum of compound **5** (CDCl_3_). |
| S39 | ROESY spectrum of compound **5** (CDCl_3_). |
| S40 | HREIMS spectrum of compound **5** |
| S41 | IR spectrum of compound **5** |

**S1 Bioassay**

**S1.1 Cytotoxicity Assay**

The cytotoxic assay was performed using the MTT method, as previous method with slight modification. Briefly, human tumor cells were seeded into 96-well plates and permitted to adhere for 12 h before drug addition. For suspended cells, they were seeded immediately before drug addition with an initial density of 5 × 10^4^ cells/ml. Each cell line was incubated with different concentrations of the compounds for 48 h. DDP and Taxol were used as positive controls. Cell viability was measured and IC 50 values were calculated.

**Table S1** Cytotoxicity of compounds **1**-**5, 7, 8** against HL-60, A-549, SMMC-7721, MCF-7 and SW480 cell lines (IC_50_ *μ*M).

| **sample** | **HL-60** | **SMMC-7721** | **A-549** | **MCF-7** | **SW480** |
| --- | --- | --- | --- | --- | --- |
| **1** | >40 | >40 | >40 | >40 | >40 |
| **2** | >40 | >40 | >40 | >40 | >40 |
| **3** | >40 | >40 | >40 | >40 | >40 |
| **4** | >40 | >40 | >40 | >40 | >40 |
| **5** | >40 | >40 | >40 | >40 | >40 |
| **7** | >40 | >40 | >40 | >40 | >40 |
| **8** | >40 | >40 | >40 | >40 | >40 |
| **DDP(MW300)** | 1.93 | 11.83 | 12.40 | 18.34 | 18.10 |
| **Taxol** | <0.008 | <0.008 | <0.008 | <0.008 | <0.008 |

**Figure S2.** ^1^H NMR spectrum of compound **1** (CD_3_OD)


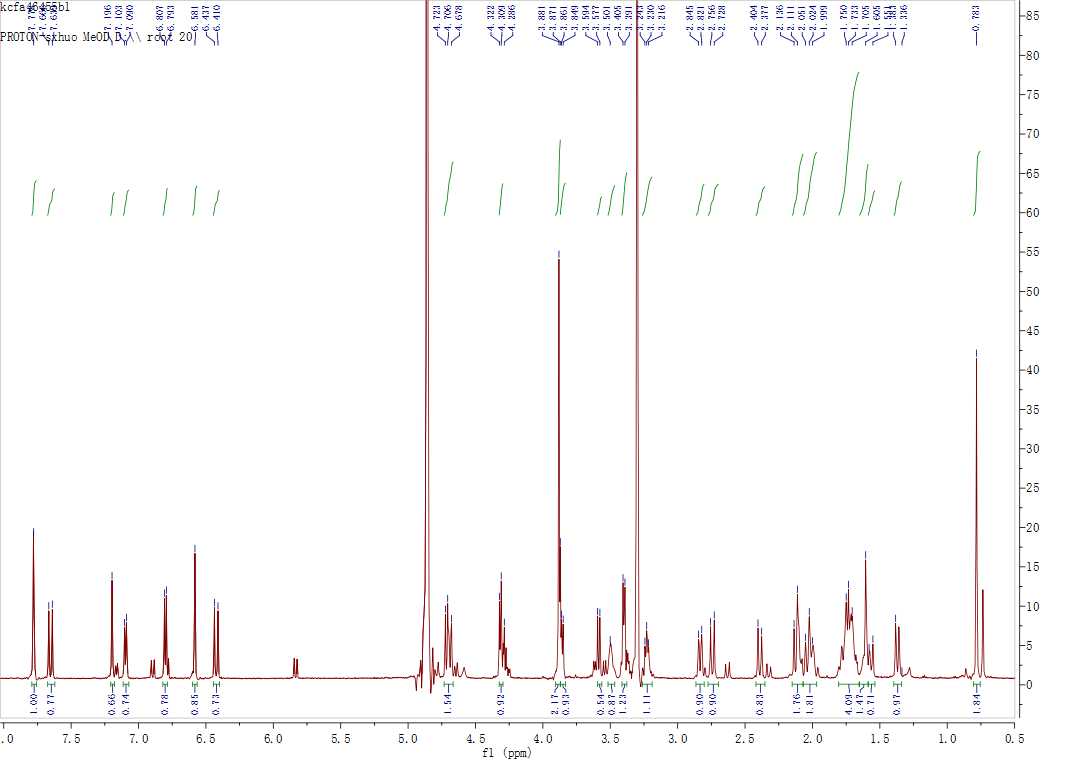


**Figure S3.** ^13^C NMR and DEPT spectra of compound **1** (CD_3_OD)


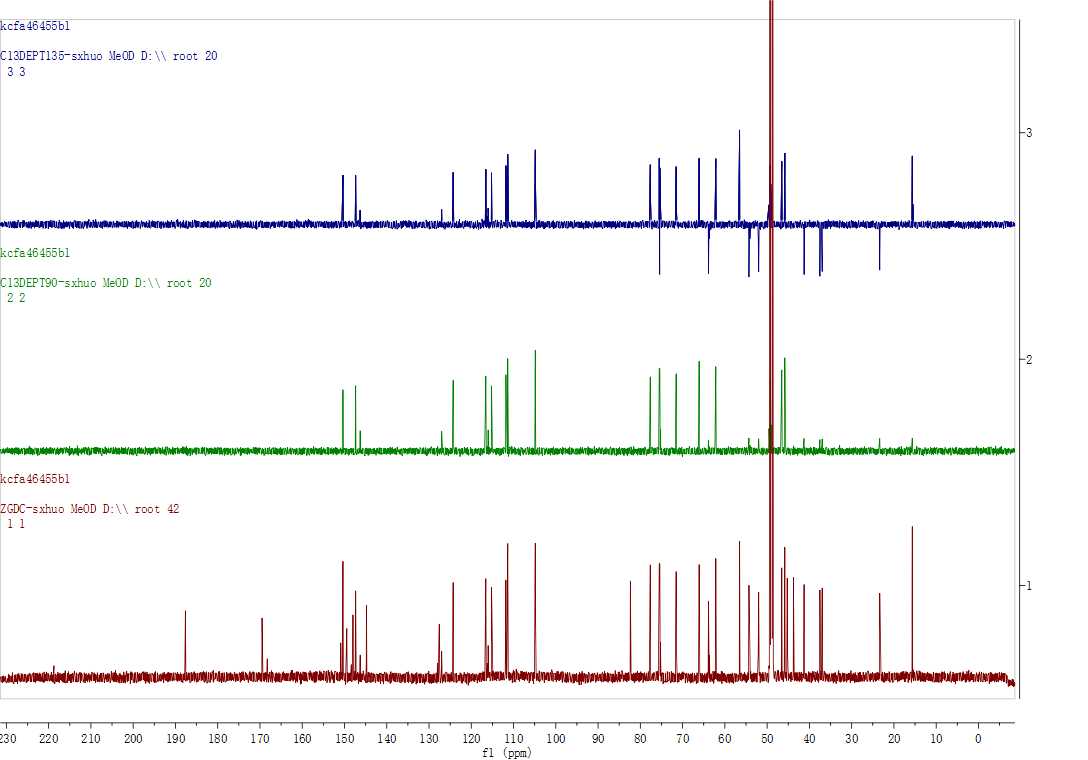

**Figure S4.** HSQC spectrum of compound **1** (CD_3_OD )


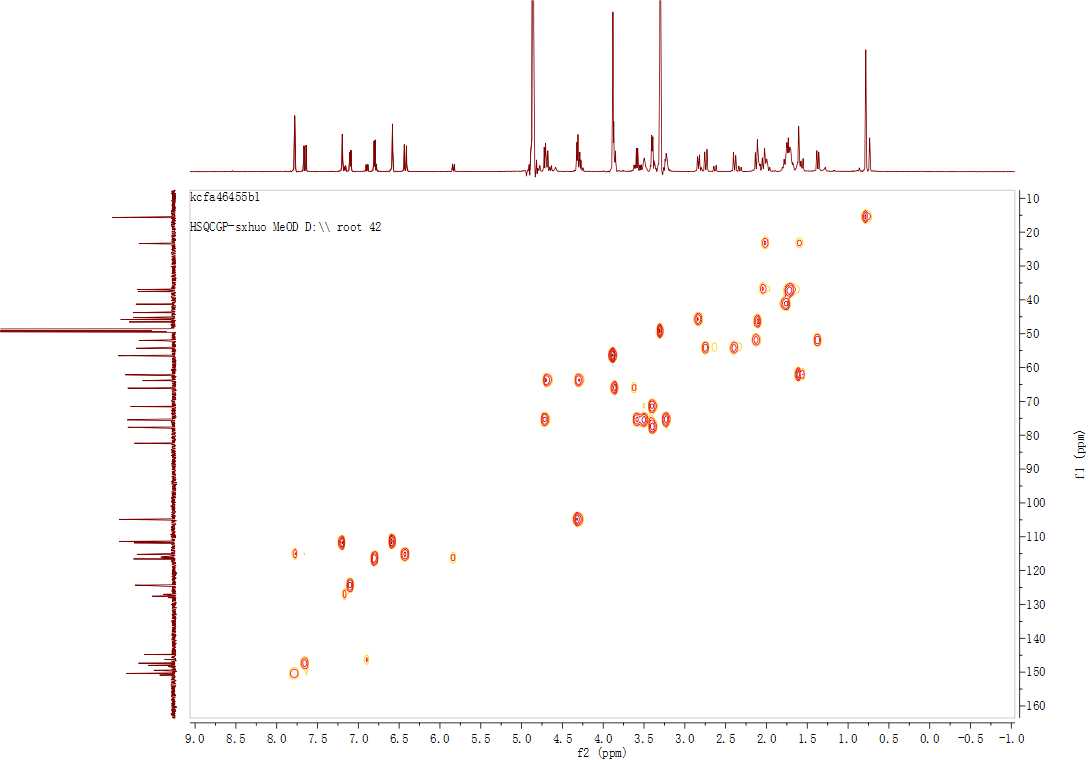


**Figure S5.** ^1^H-^1^H COSY spectrum of compound **1** (CD_3_OD )


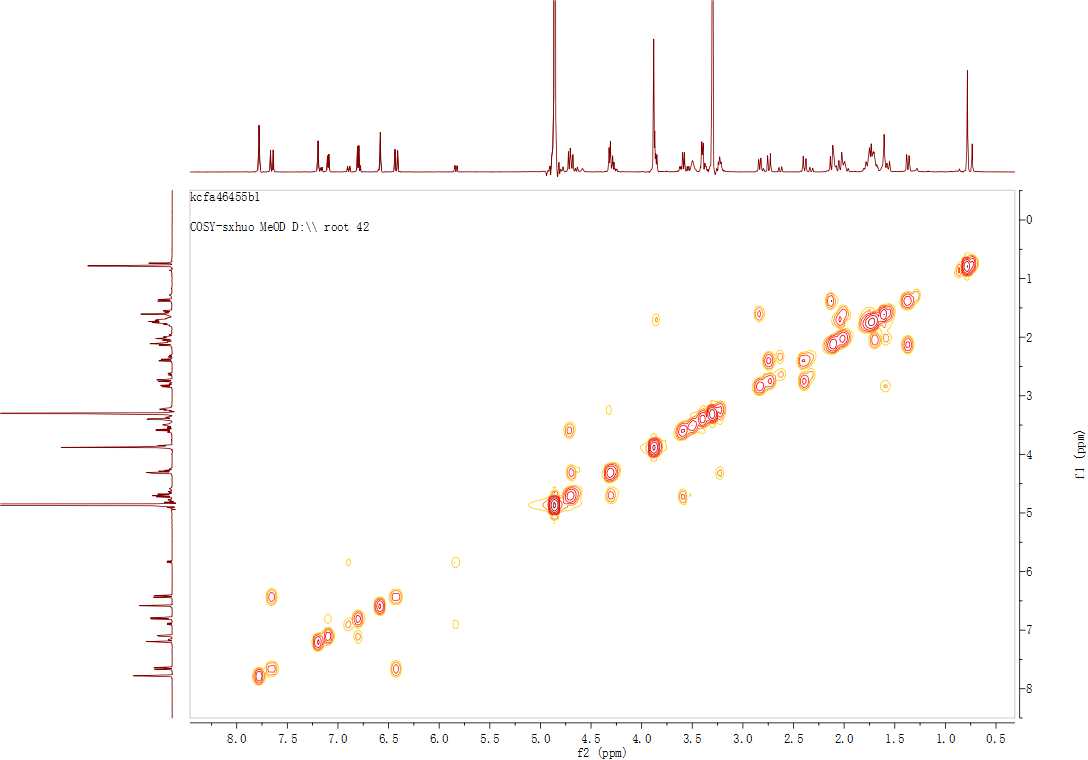


**Figure S6.** HMBC spectrum of compound **1** (CD_3_OD)


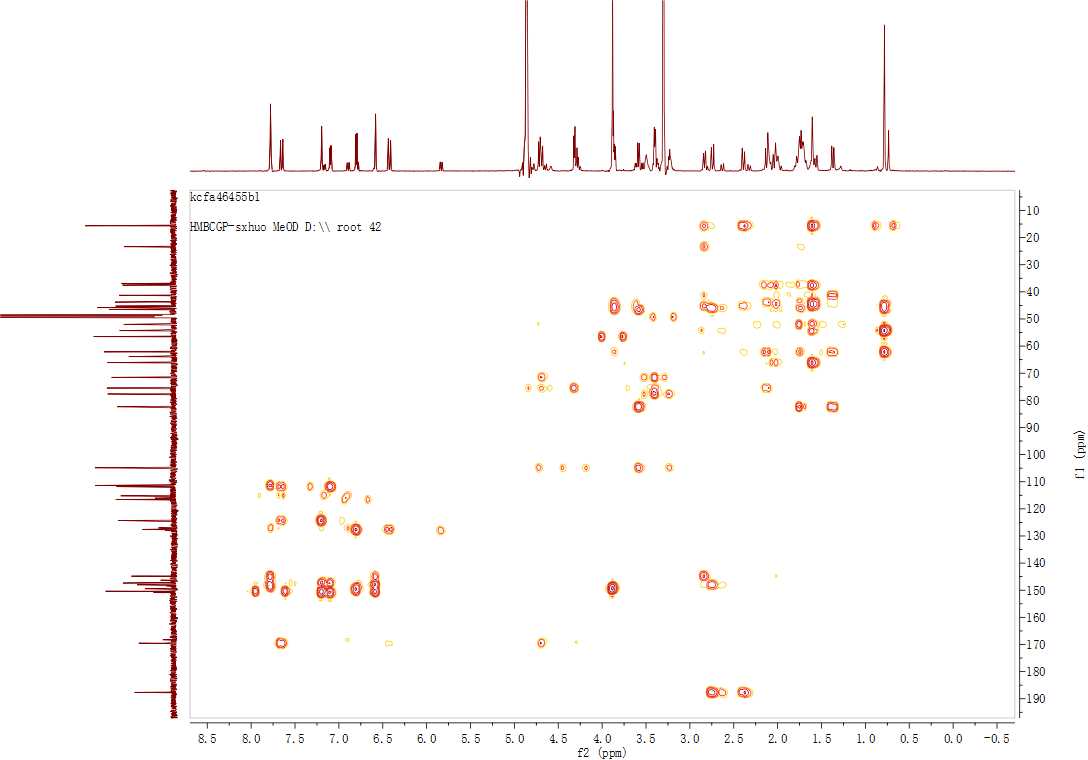


**Figure S7.** ROESY spectrum of compound **1** (CD_3_OD )


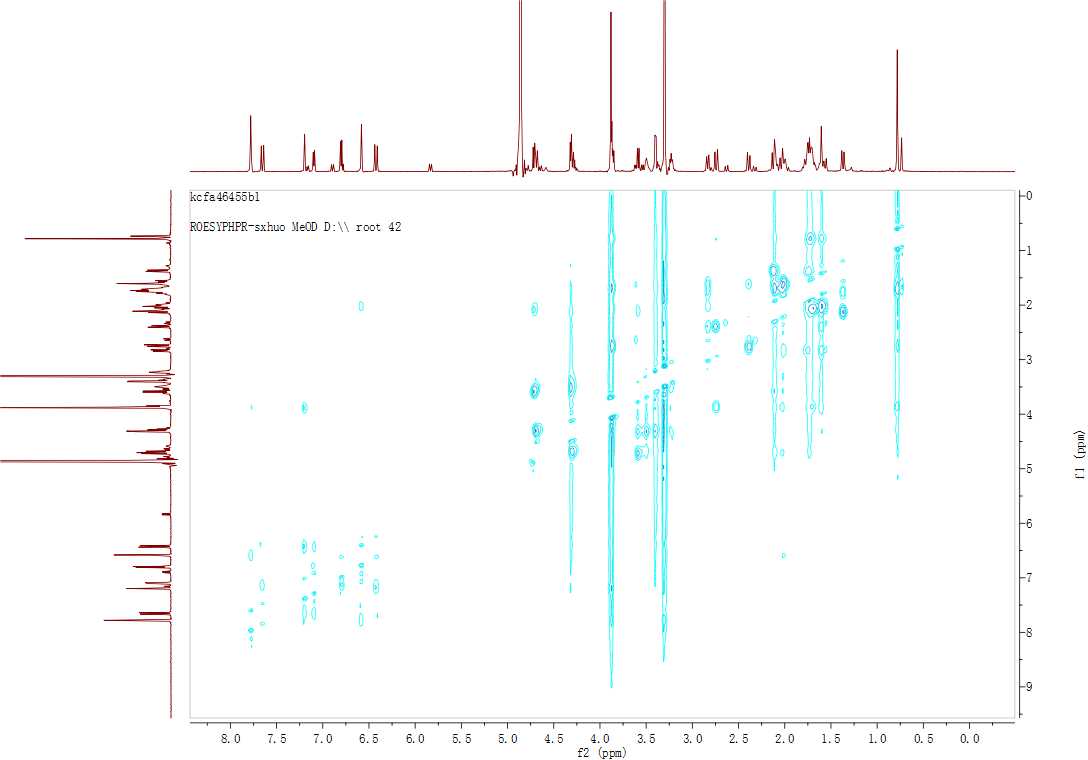


**Figure S8.** HREIMS spectrum of compound **1**

**
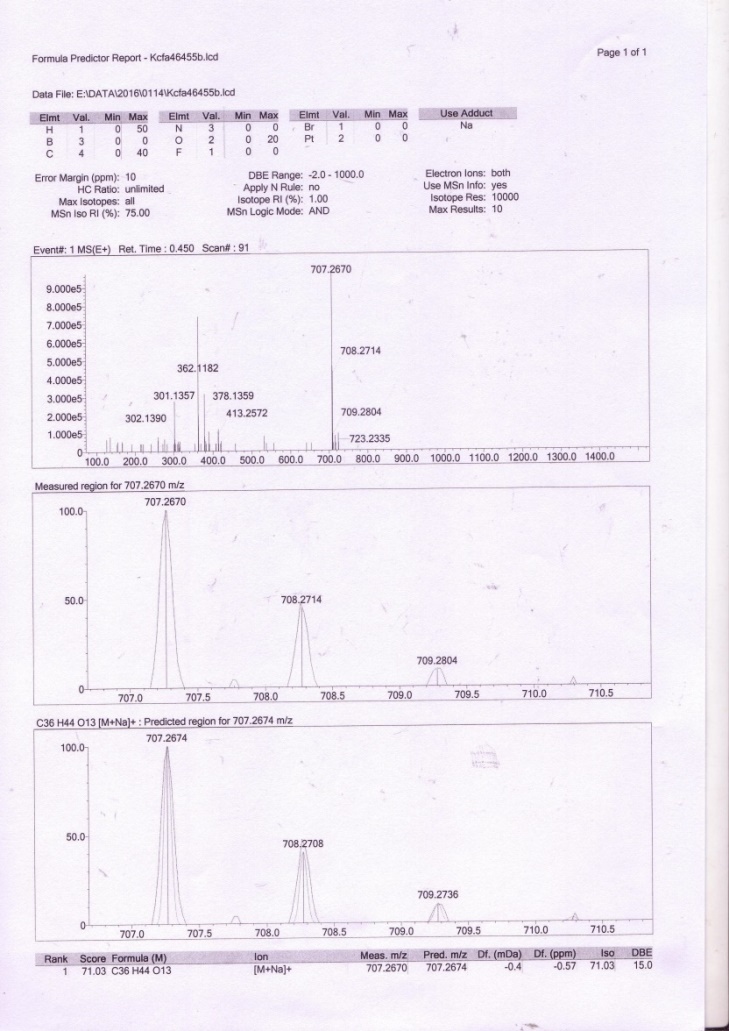
**

**Figure S9.** IR spectrum of compound **1**

**
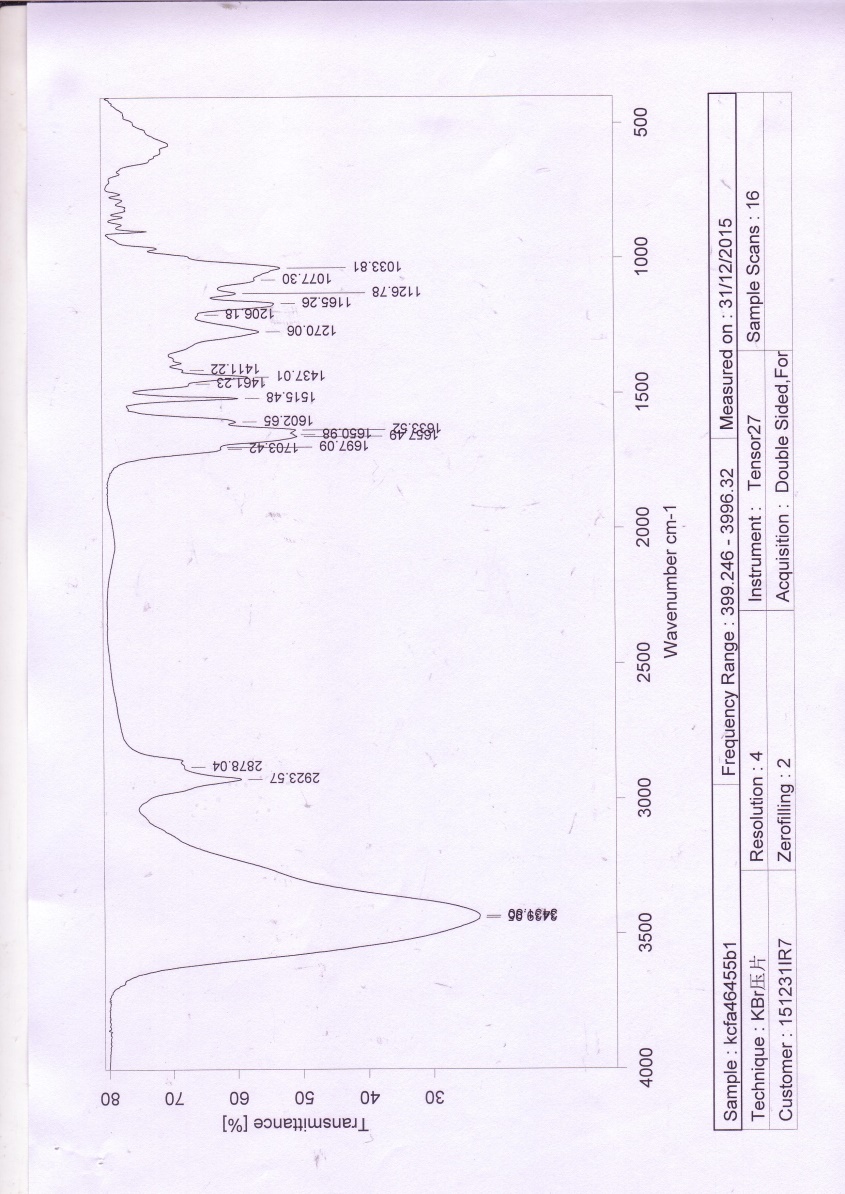
**

**Figure S10.** ^1^H NMR spectrum of compound **2** (CD_3_OD)


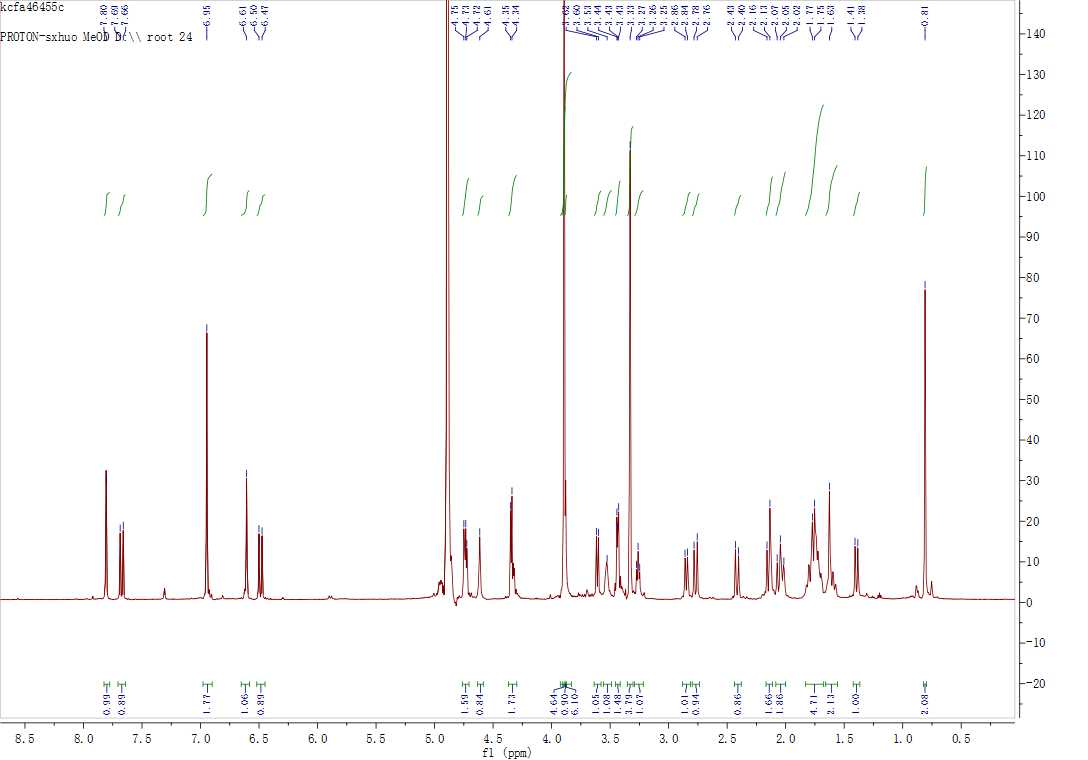


**Figure S11.** ^13^C NMR and DEPT spectra of compound **2** (CD_3_OD)


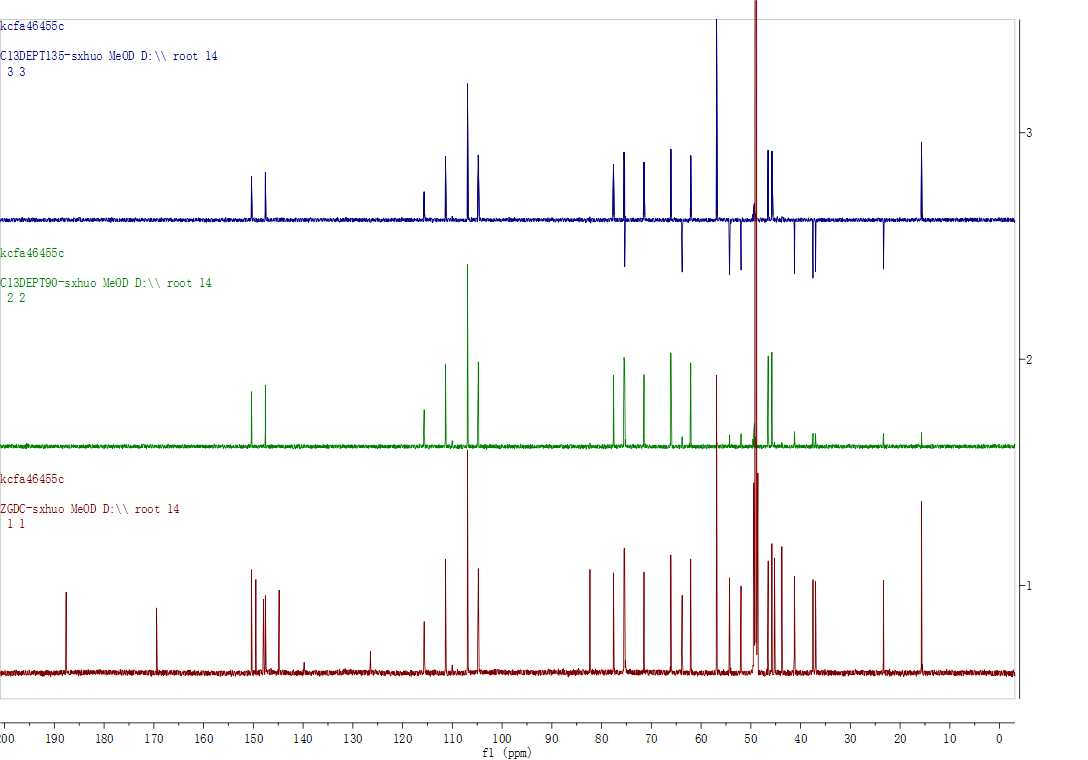

**Figure S12.** HSQC spectrum of compound **2** (CD_3_OD )


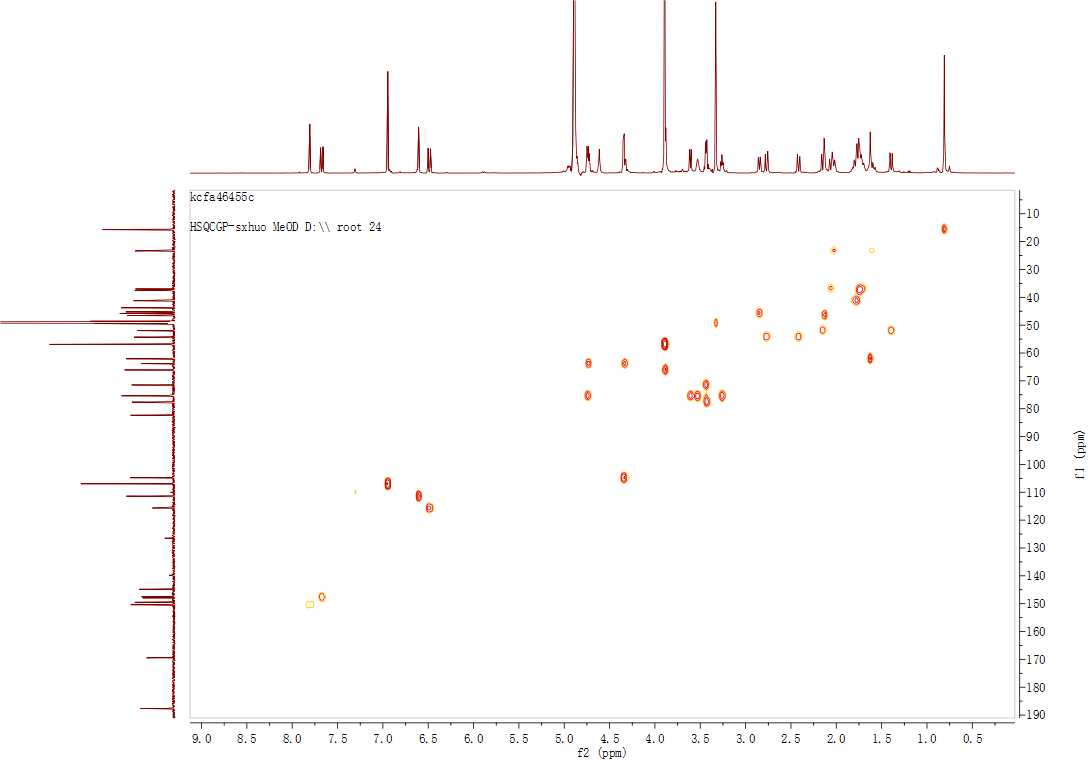


**Figure S13.** ^1^H-^1^H COSY spectrum of compound **2** (CD_3_OD )


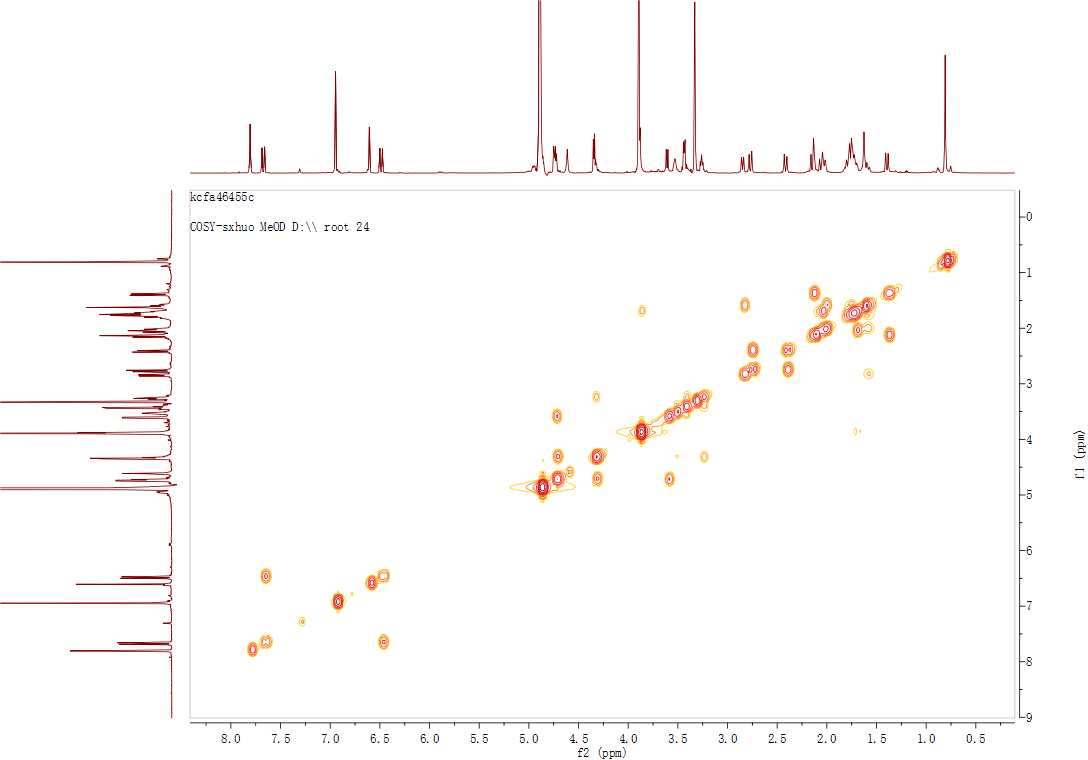


**Figure S14.** HMBC spectrum of compound **2** (CD_3_OD)


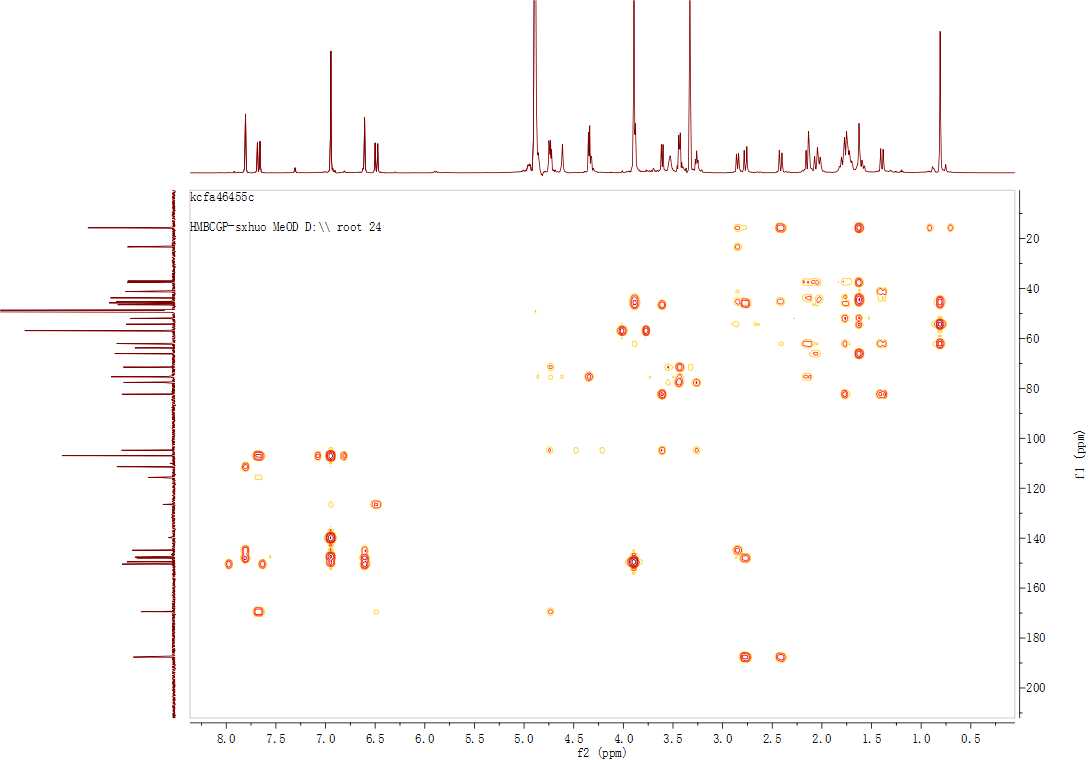


**Figure S15.** ROESY spectrum of compound **2** (CD_3_OD )


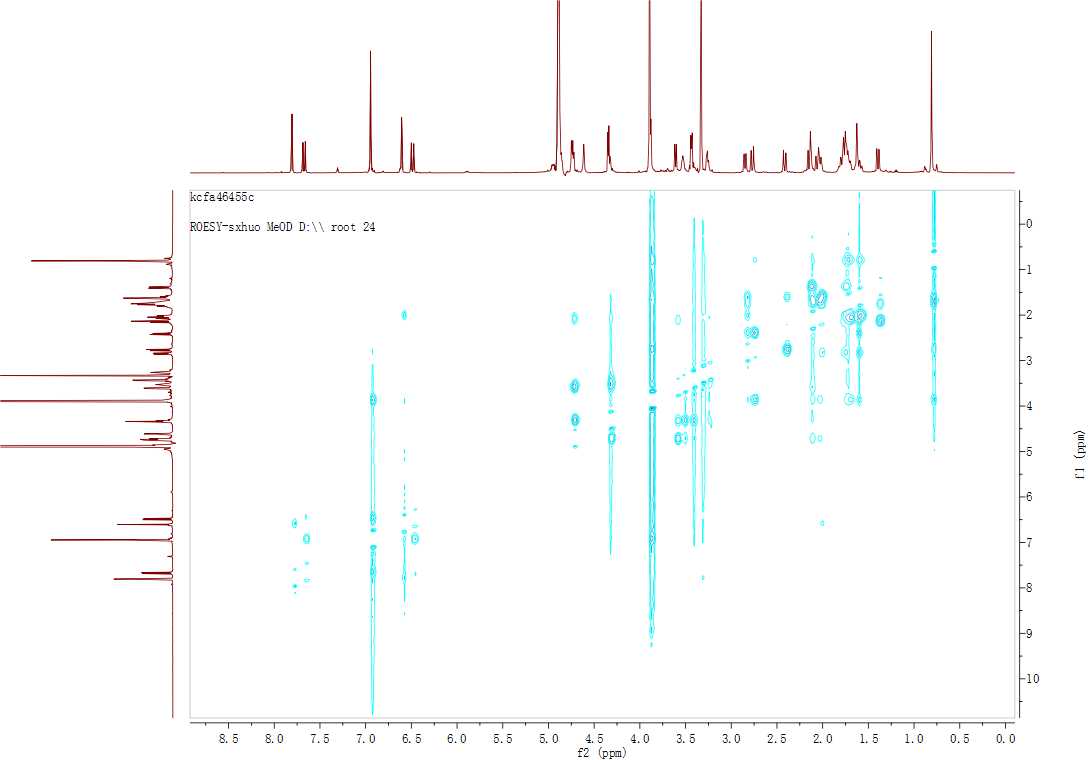


**Figure S16.** HREIMS spectrum of compound **2**


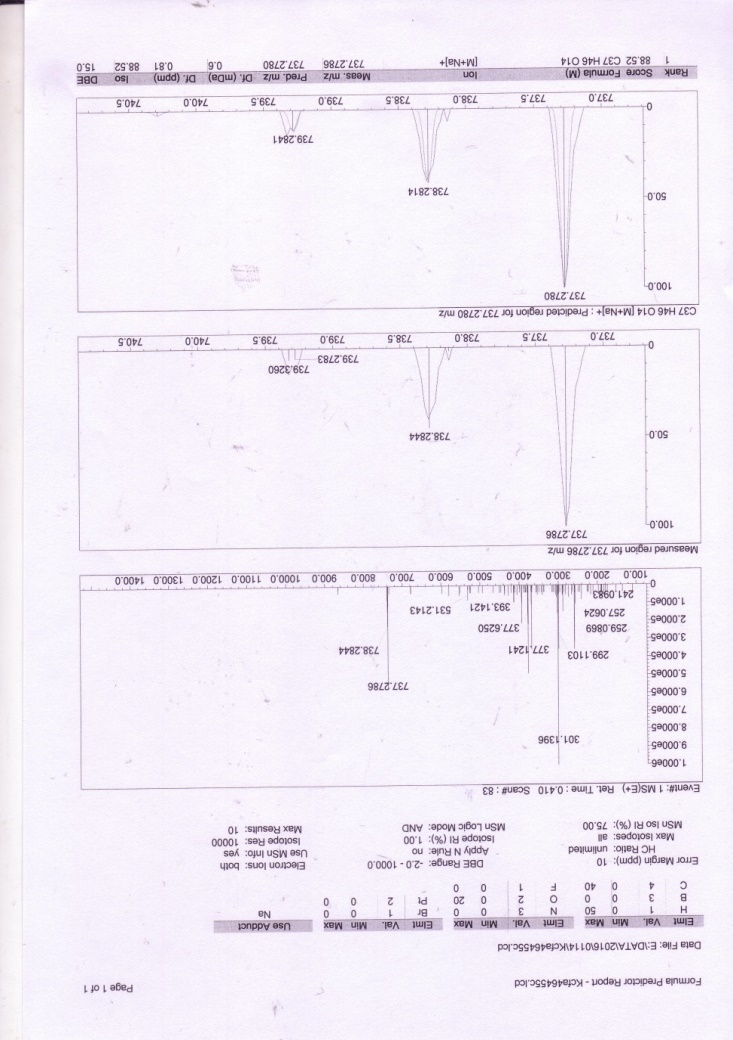


**Figure S17.** IR spectrum of compound **2**

**
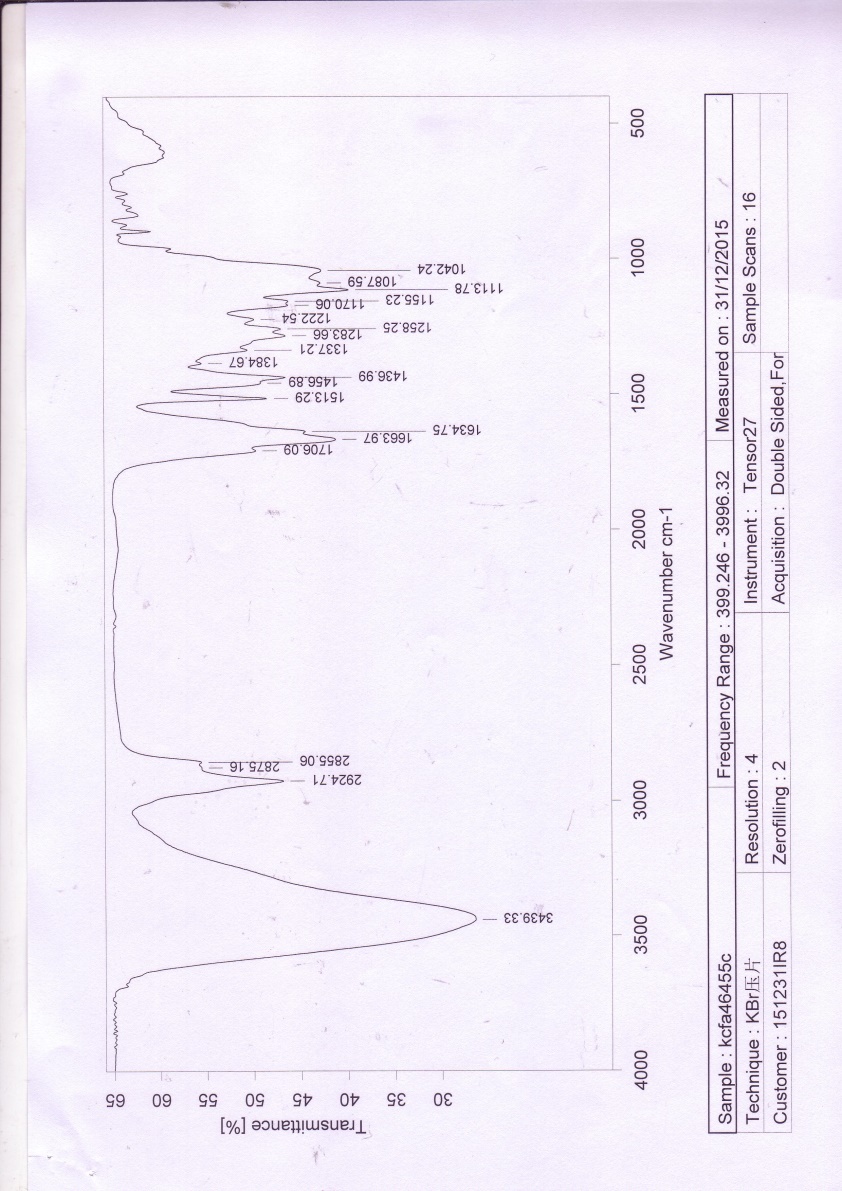
**

**Figure S18.** ^1^H NMR spectrum of compound **3** (CDCl_3_)


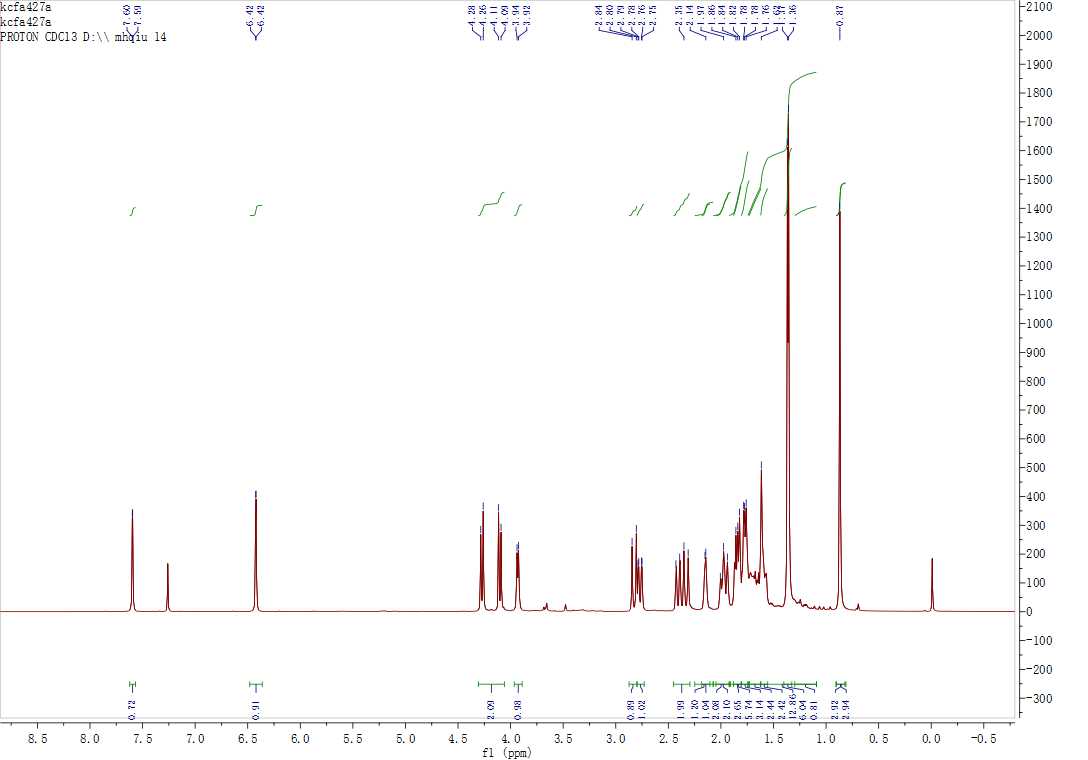


**Figure S19.** ^13^C NMR and DEPT spectra of compound **3** (CDCl_3_)


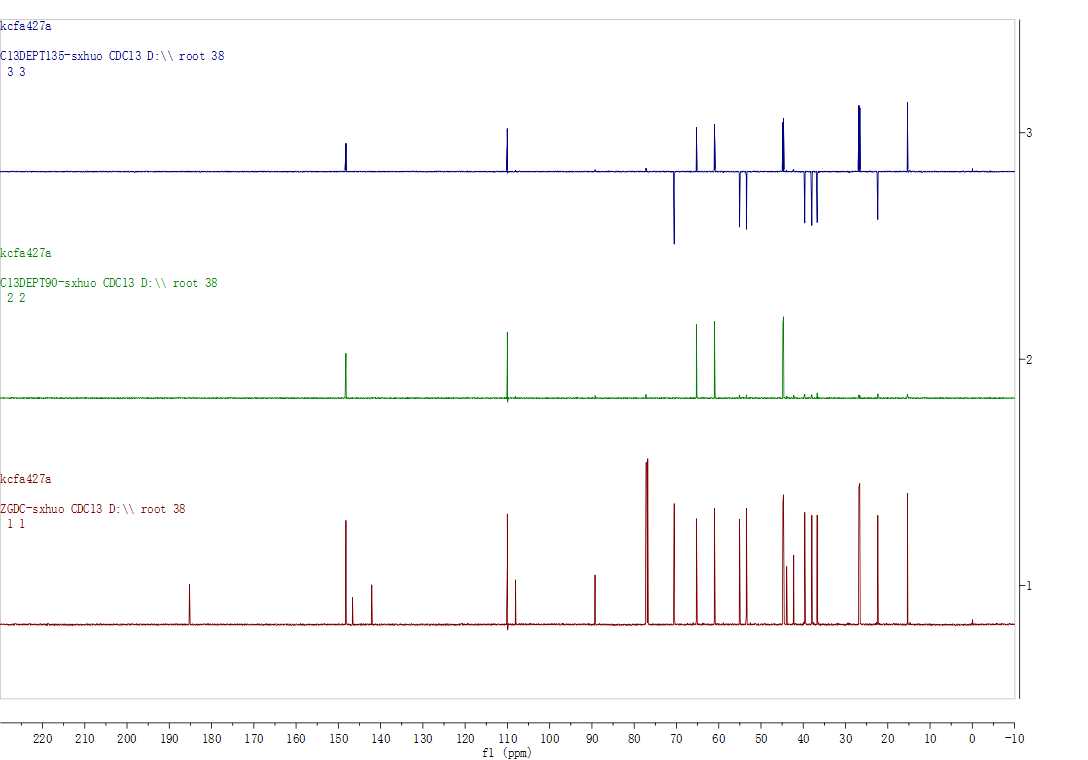

**Figure S20.** HSQC spectrum of compound **3** (CDCl_3_)


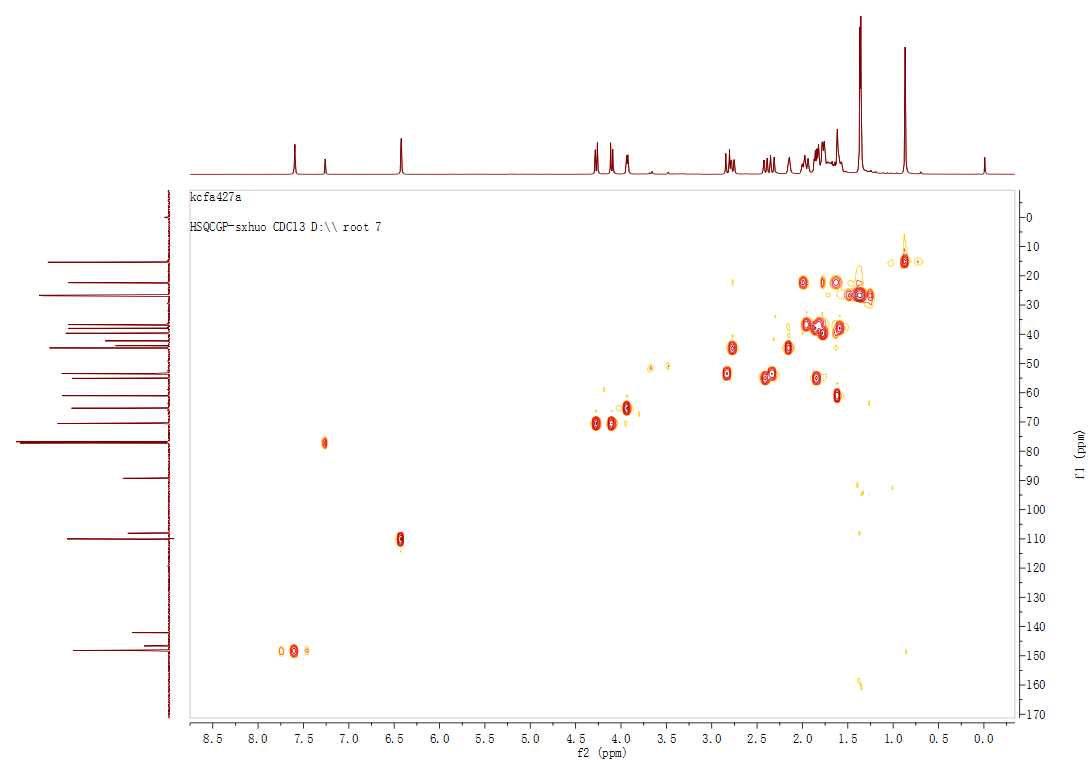


**Figure S21.** ^1^H-^1^H COSY spectrum of compound **3** (CDCl_3_)


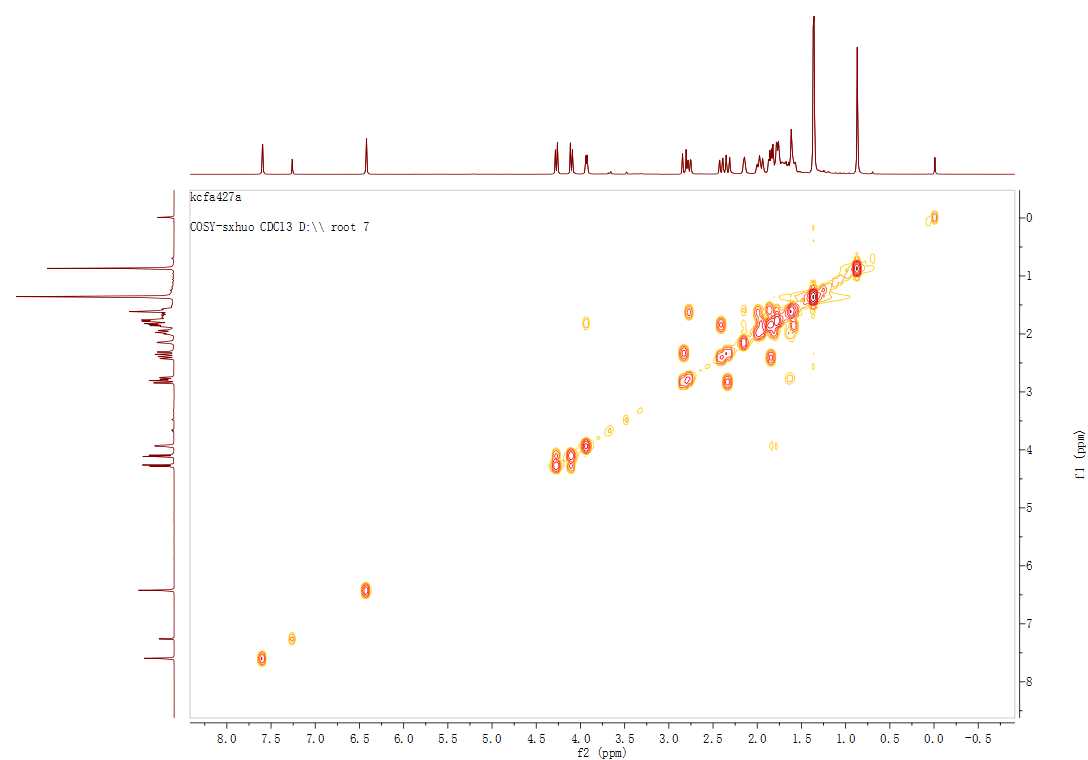


**Figure S22.** HMBC spectrum of compound **3** (CDCl_3_)


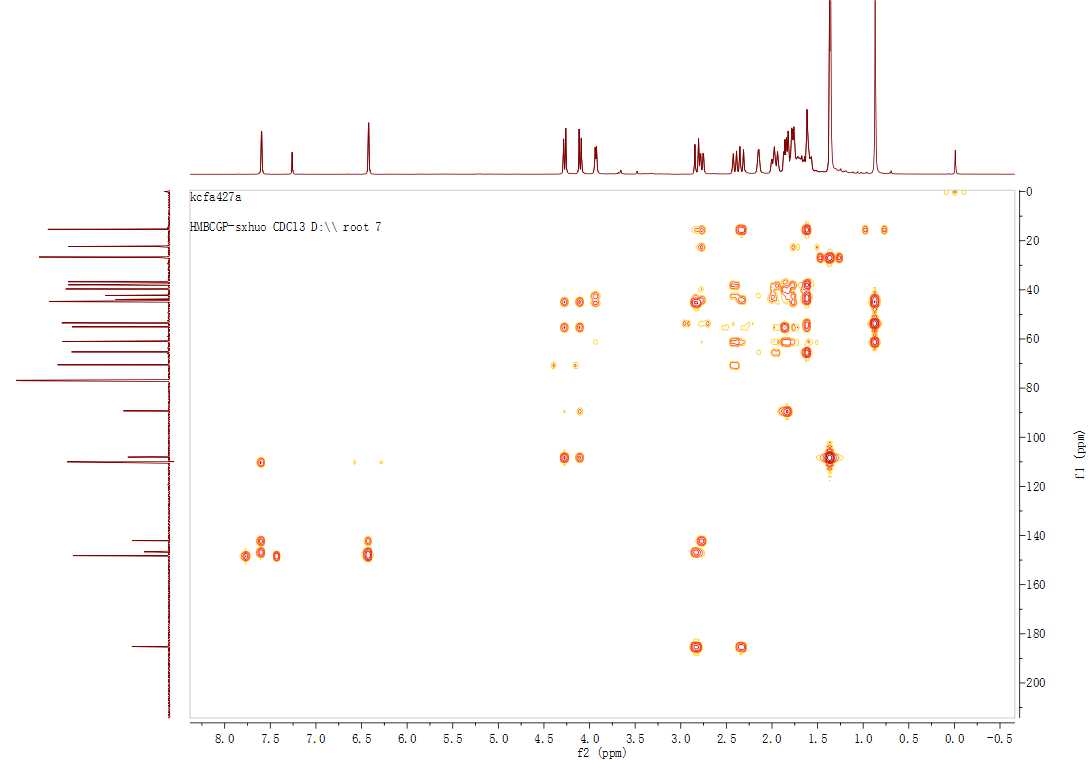


**Figure S23.** ROESY spectrum of compound **3** (CDCl_3_)


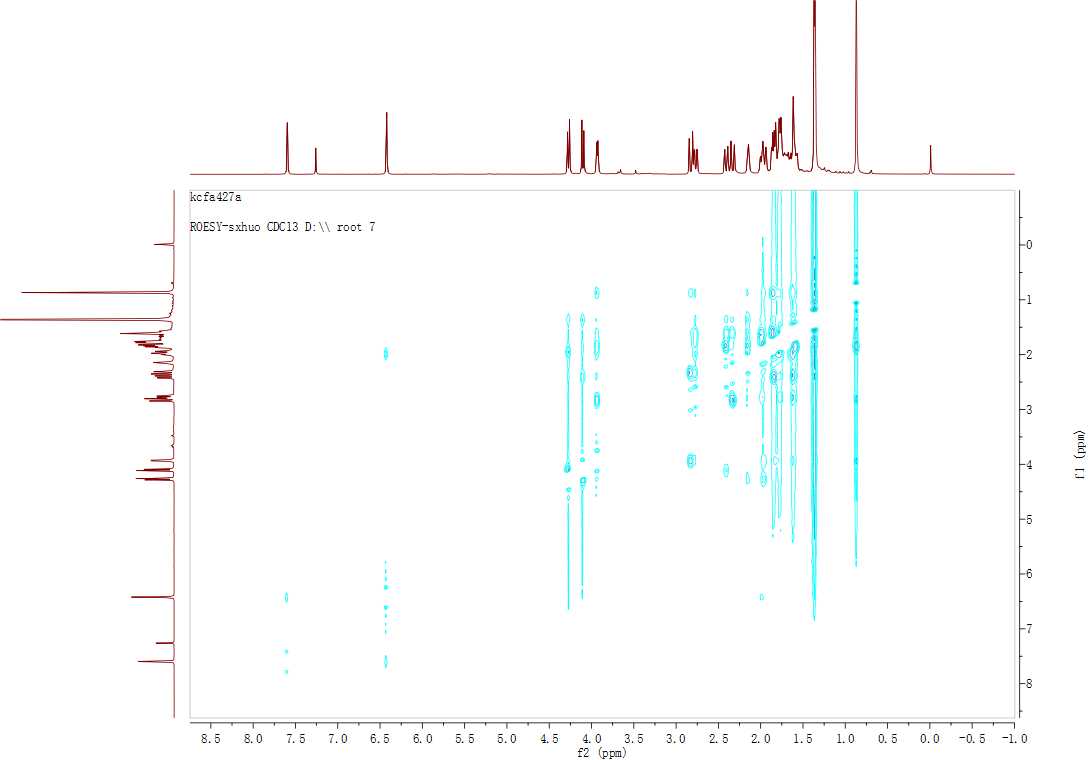


**Figure S24.** HREIMS spectrum of compound **3**


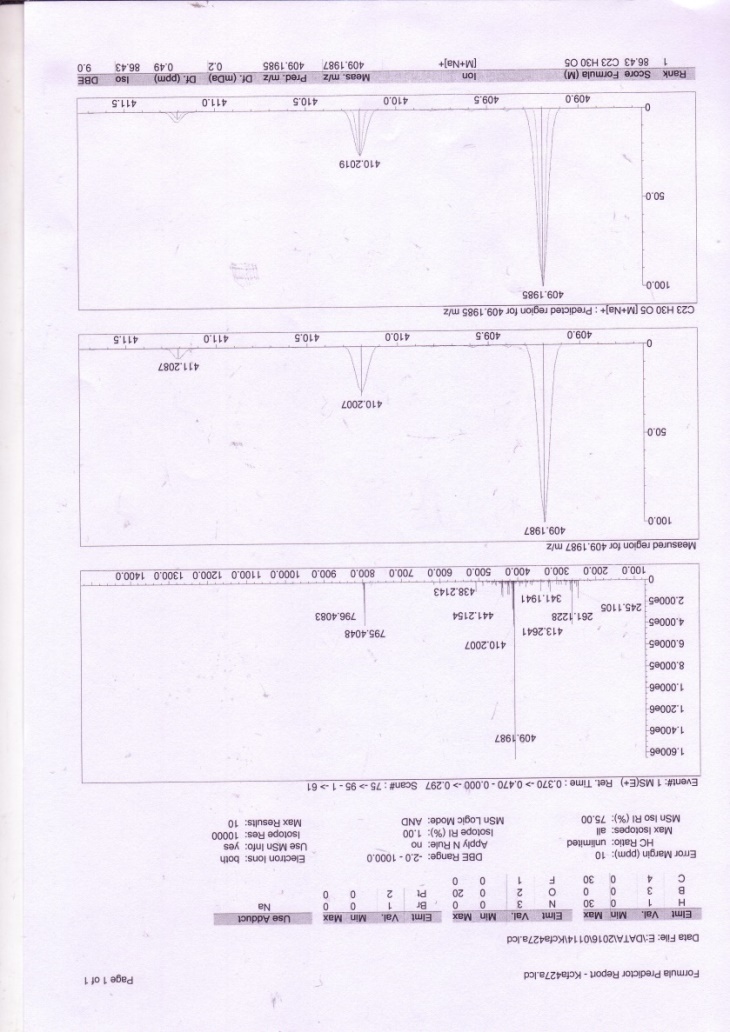


**Figure S25.** IR spectrum of compound **3**


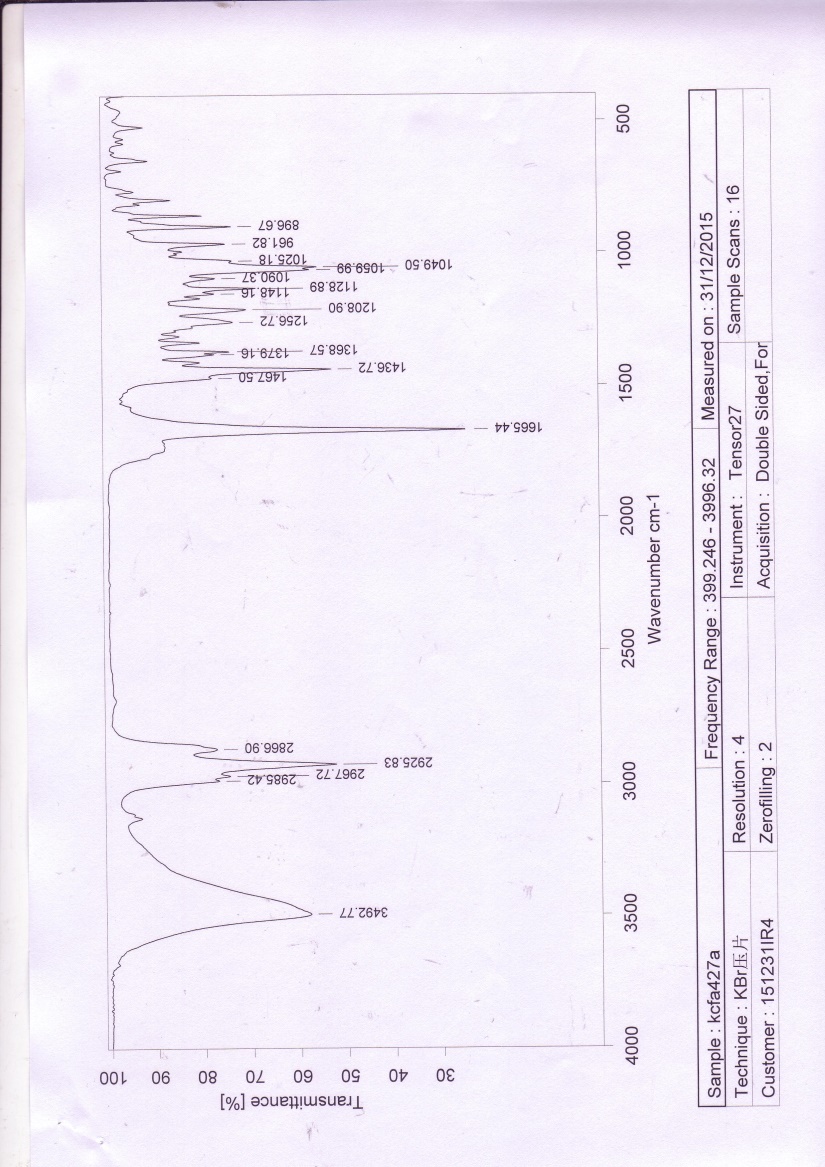


**Figure S26.** ^1^H NMR spectrum of compound **4** (CD_3_OD)


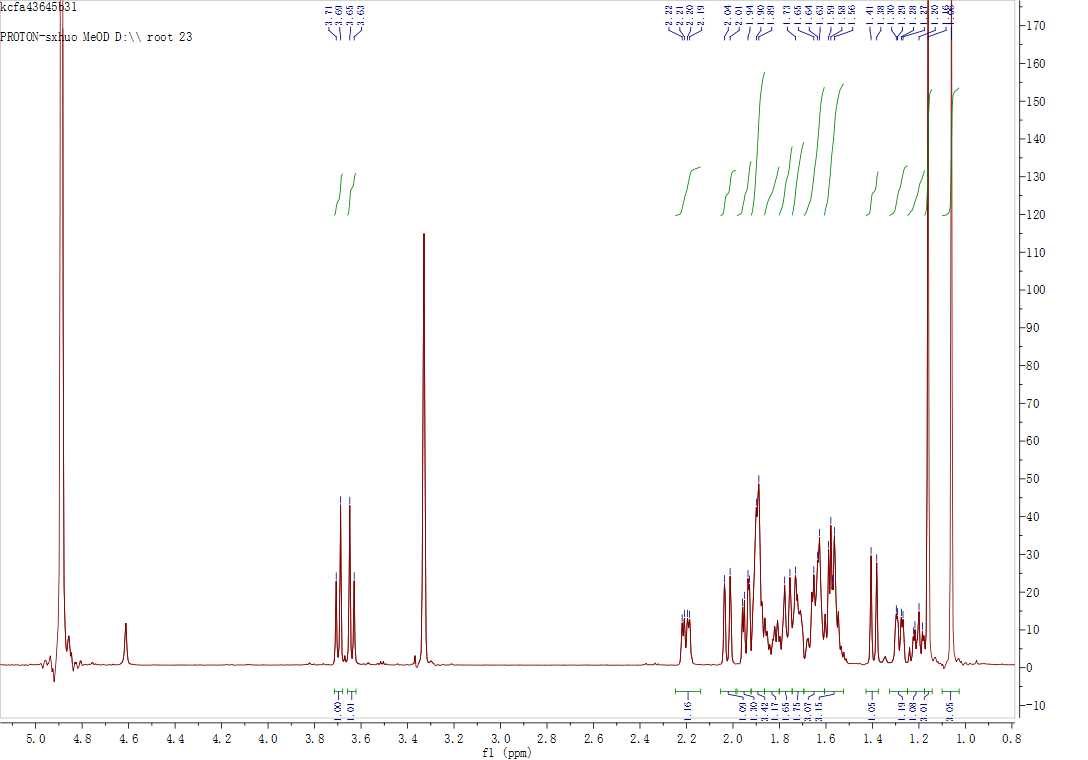


**Figure S27.** ^13^C NMR and DEPT spectra of compound **4** (CD_3_OD)


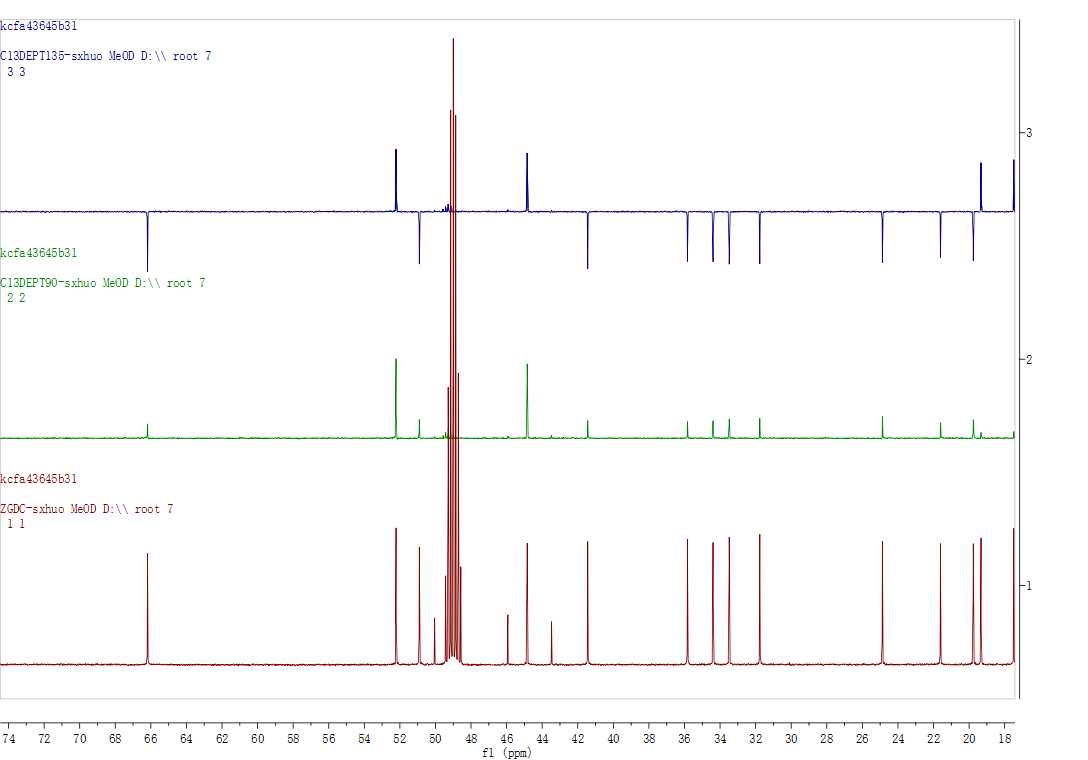

**Figure S28.** HSQC spectrum of compound **4** (CD_3_OD )


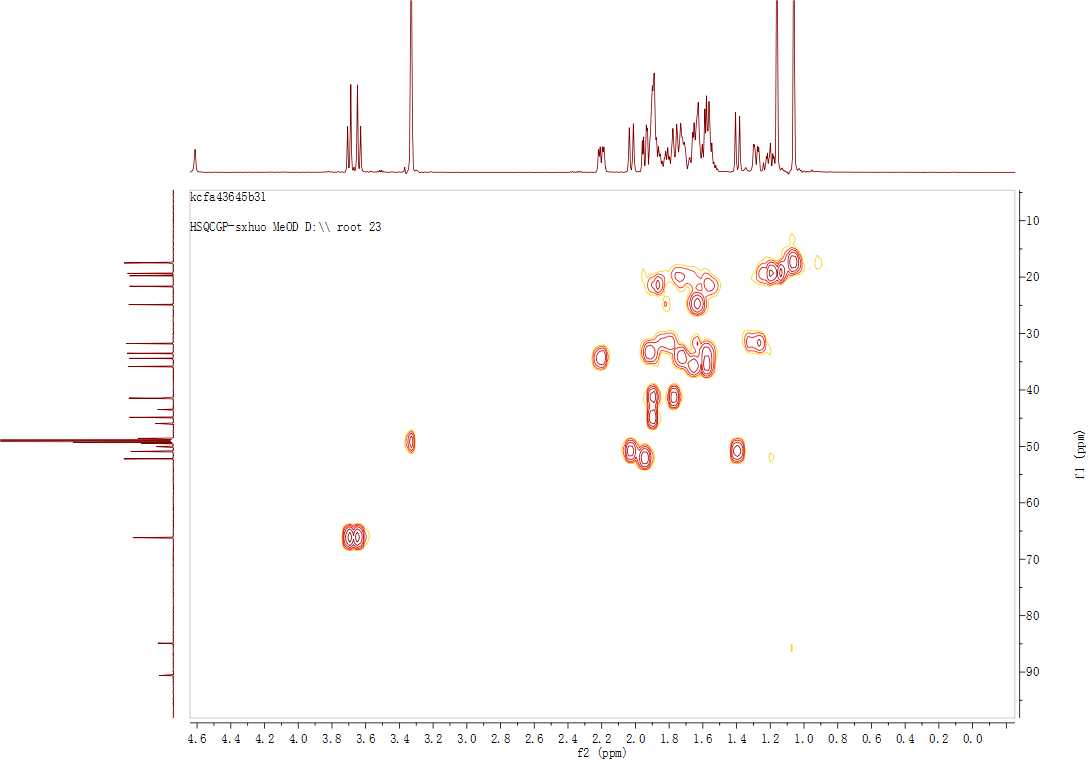


**Figure S29.** ^1^H-^1^H COSY spectrum of compound **4** (CD_3_OD )


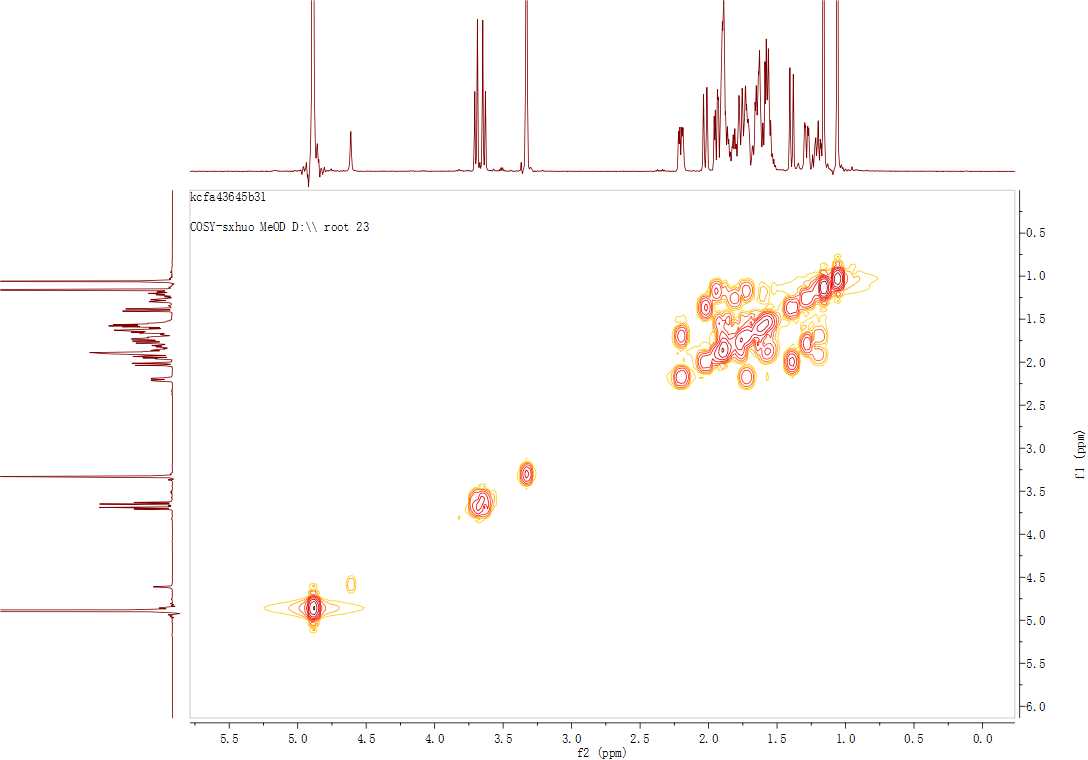


**Figure S30.** HMBC spectrum of compound **4** (CD_3_OD)


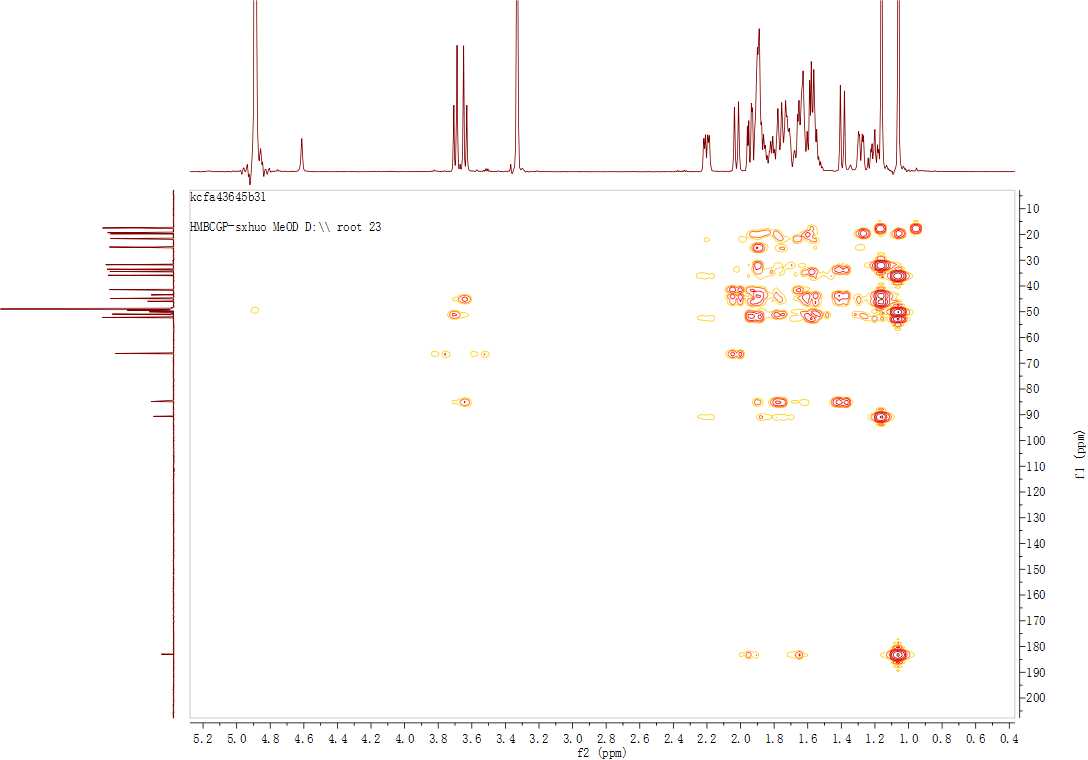


**Figure S31.** ROESY spectrum of compound **4** (CD_3_OD )


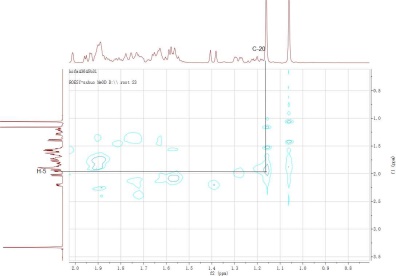

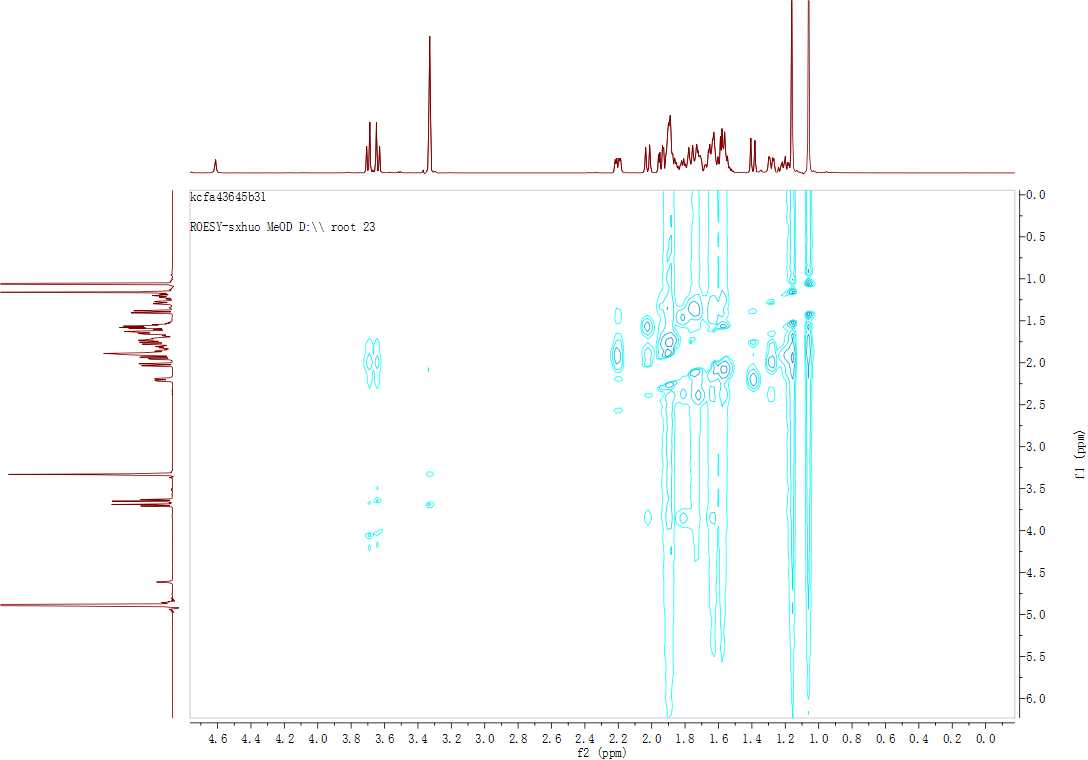


**Figure S32.** HREIMS spectrum of compound **4**

**
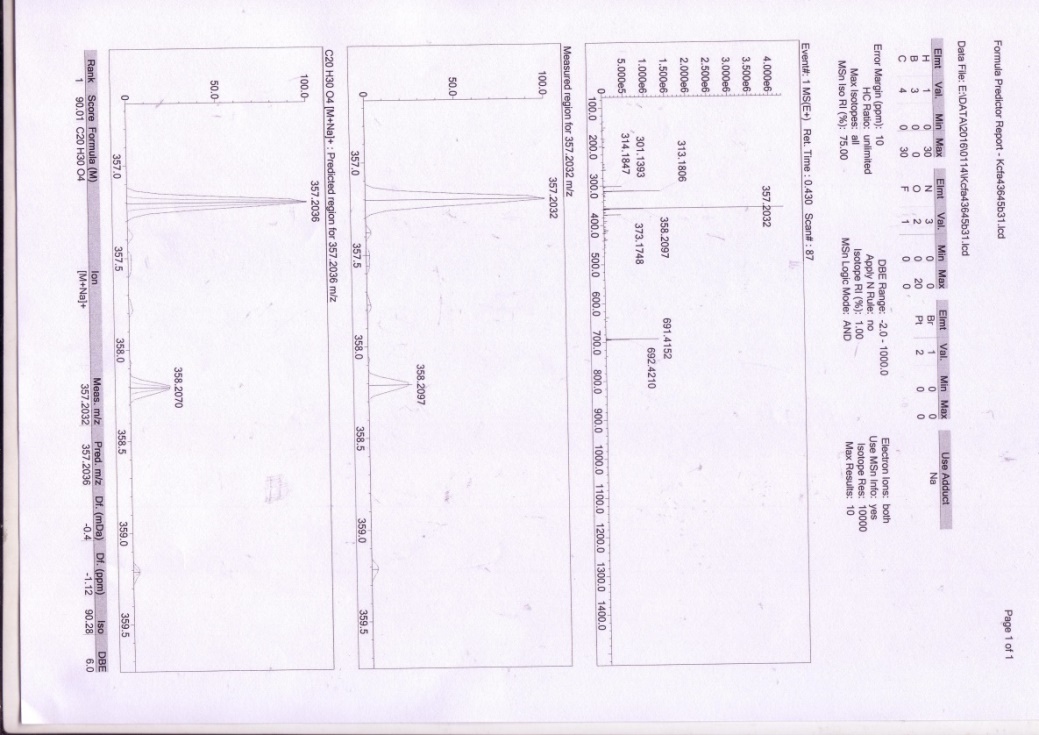
**

**Figure S33.** IR spectrum of compound **4**

**
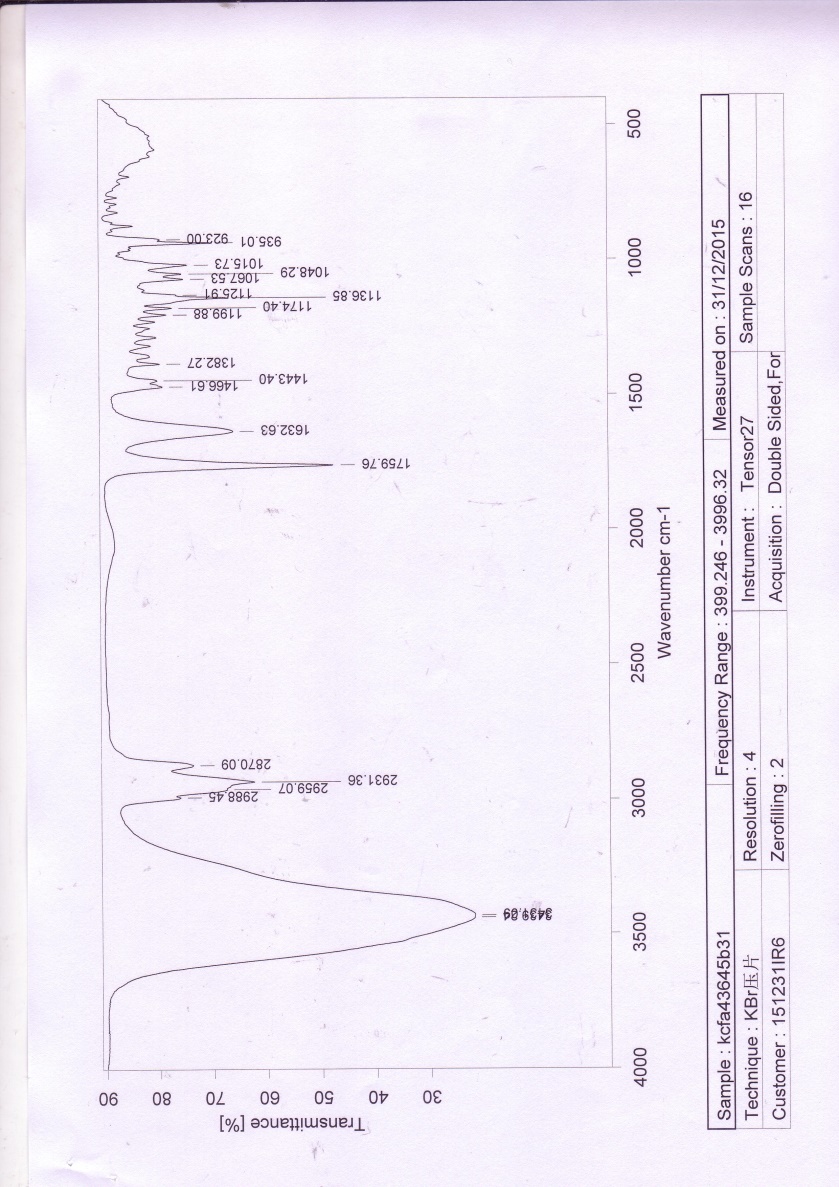
**

**Figure S34.** ^1^H NMR spectrum of compound **5** (CDCl_3_)


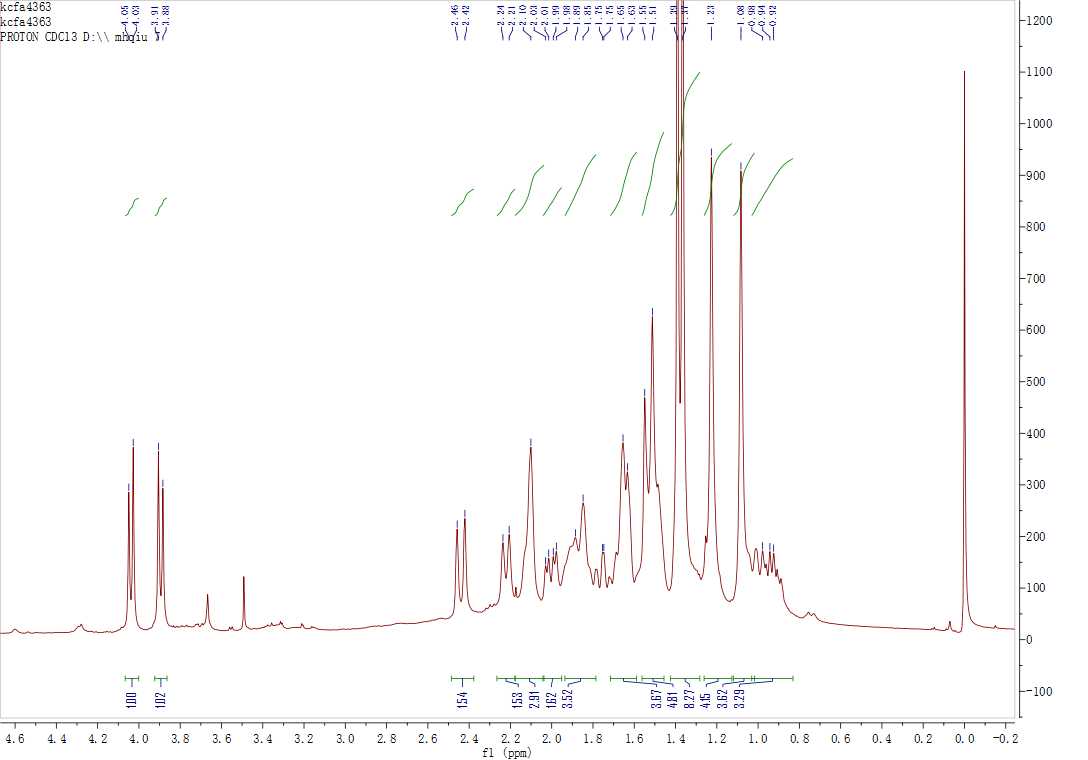


**Figure S35.** ^13^C NMR and DEPT spectra of compound **5** (CDCl_3_)


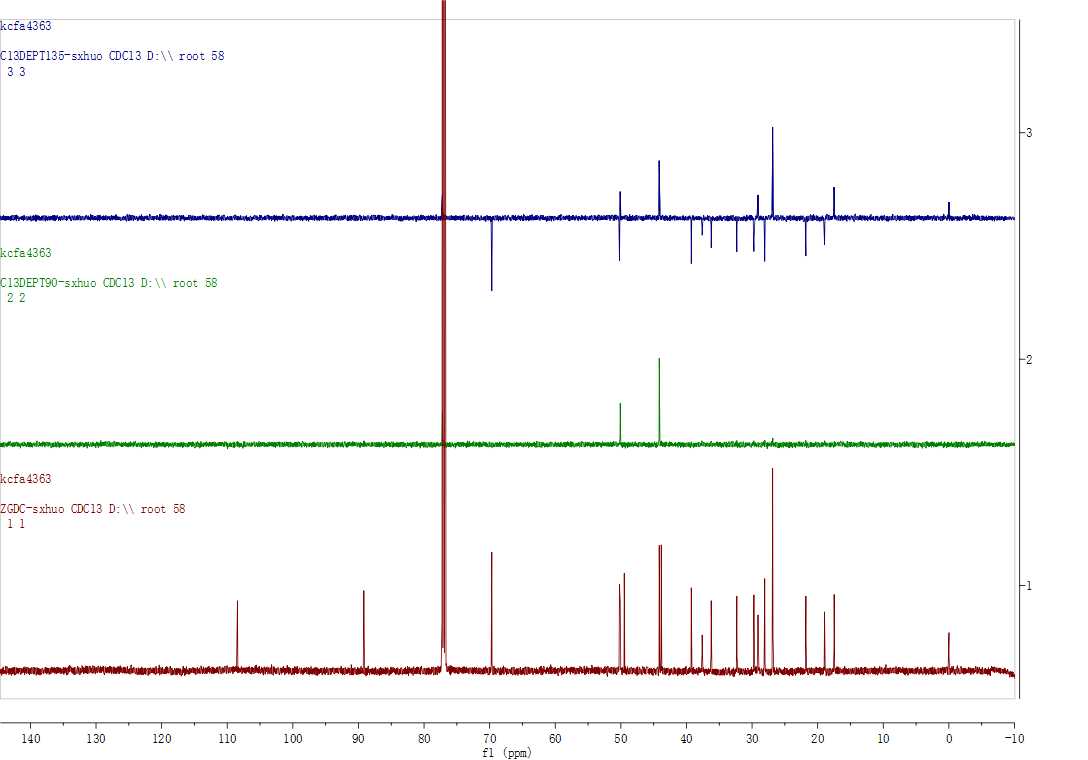

**Figure S36.** HSQC spectrum of compound **5** (CDCl_3_)


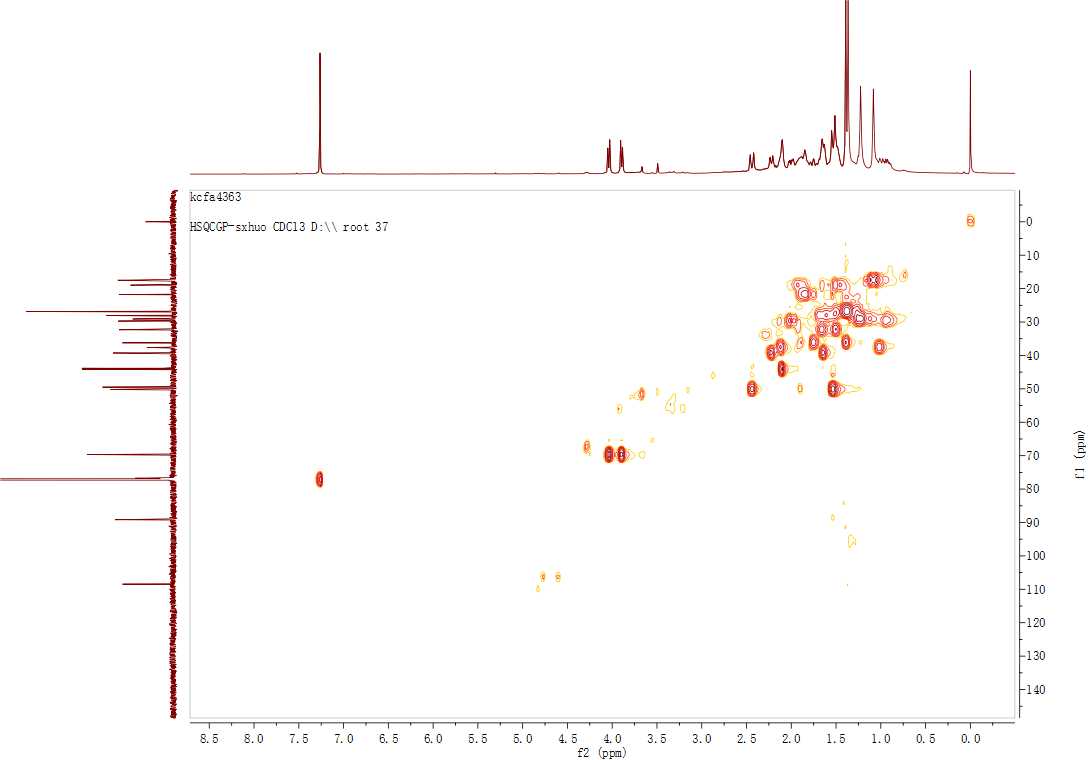


**Figure S37.** ^1^H-^1^H COSY spectrum of compound **5** (CDCl_3_)


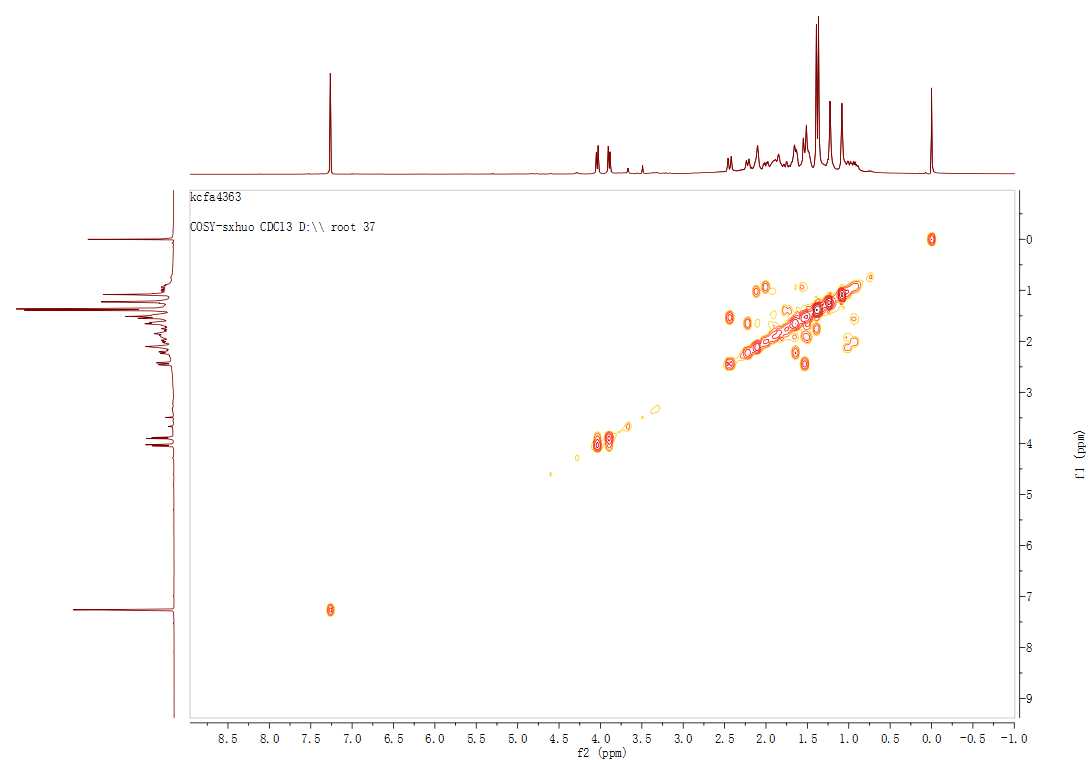


**Figure S38.** HMBC spectrum of compound **5** (CDCl_3_)

**
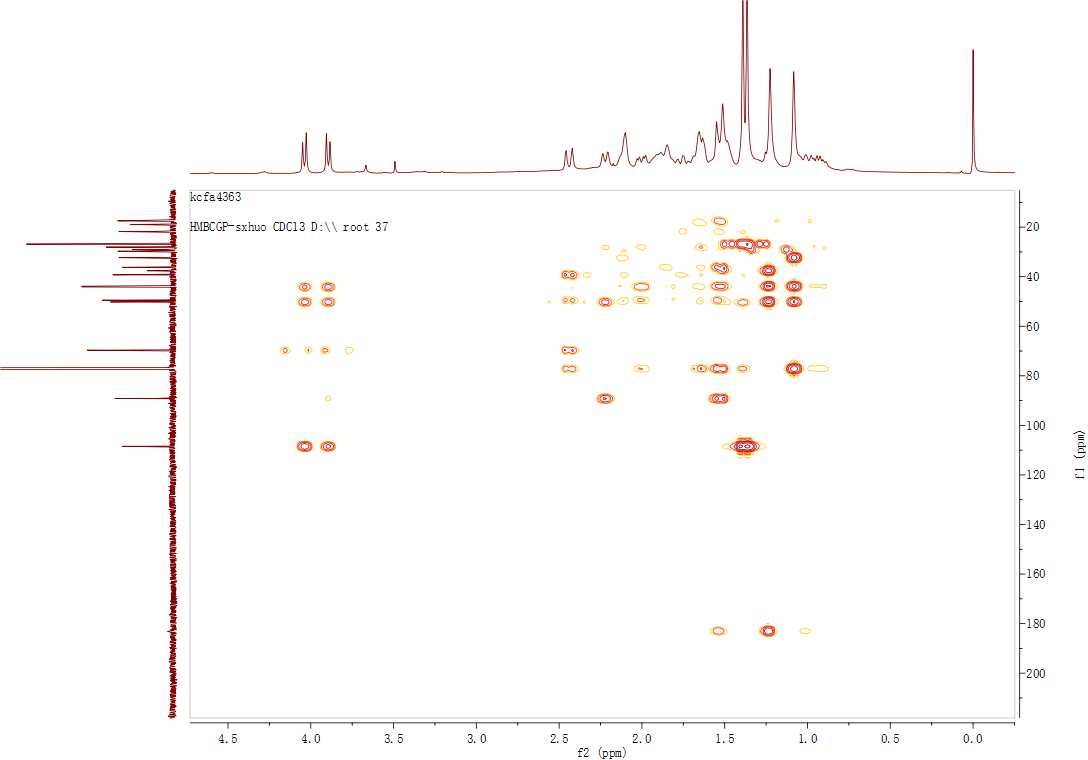
**

**Figure S39.** ROESY spectrum of compound **5** (CDCl_3_)


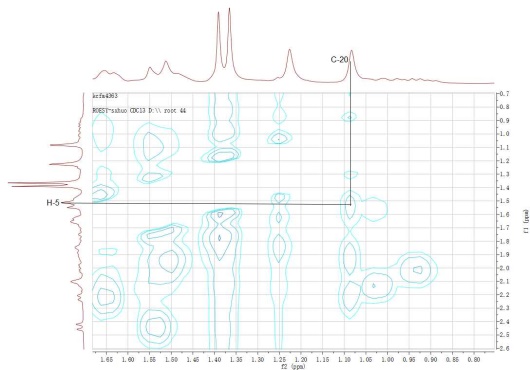

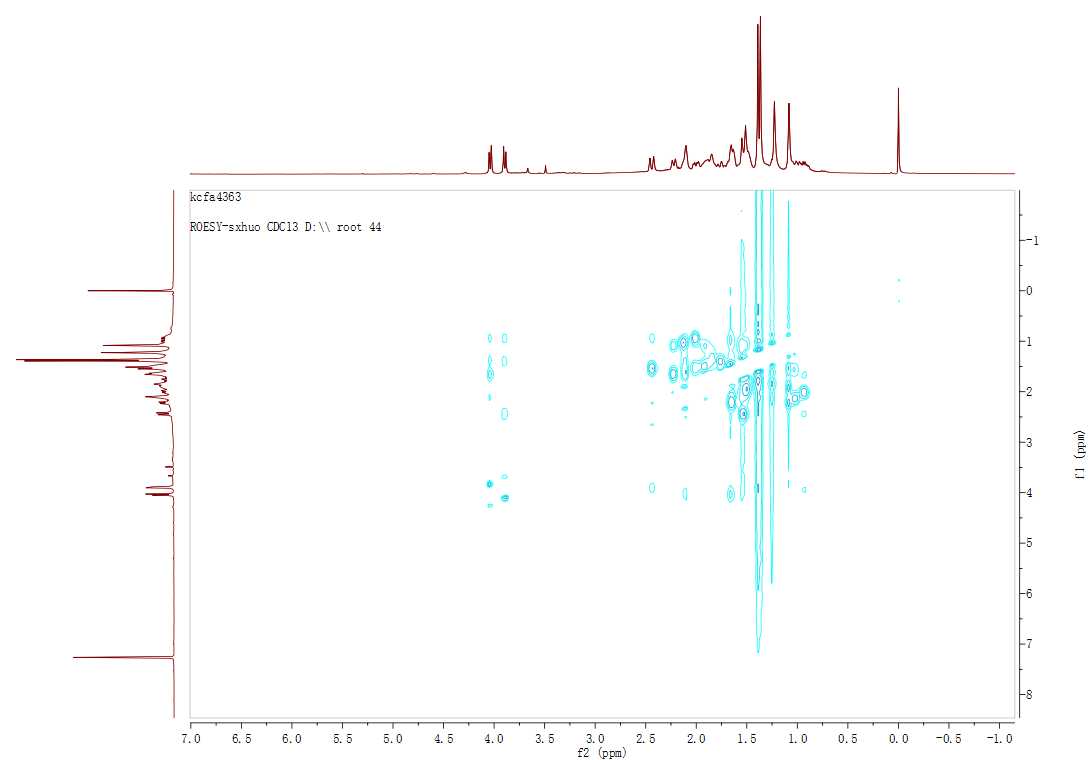


**Figure S40.** HREIMS spectrum of compound **5**


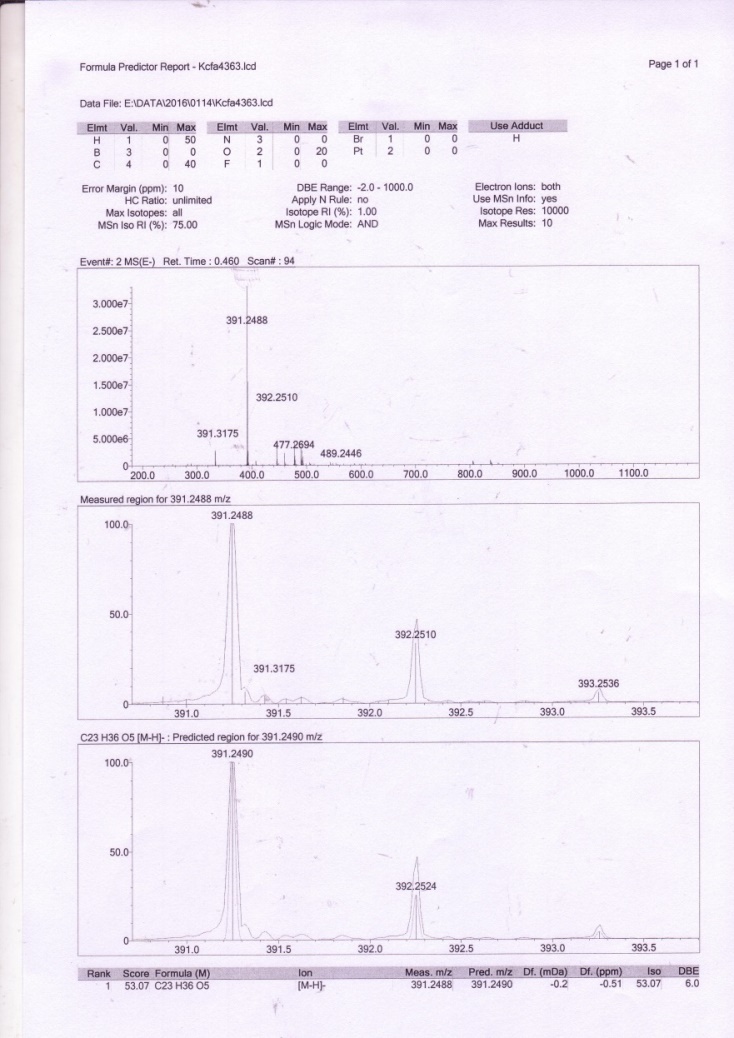


**Figure S41.** IR spectrum of compound **5**


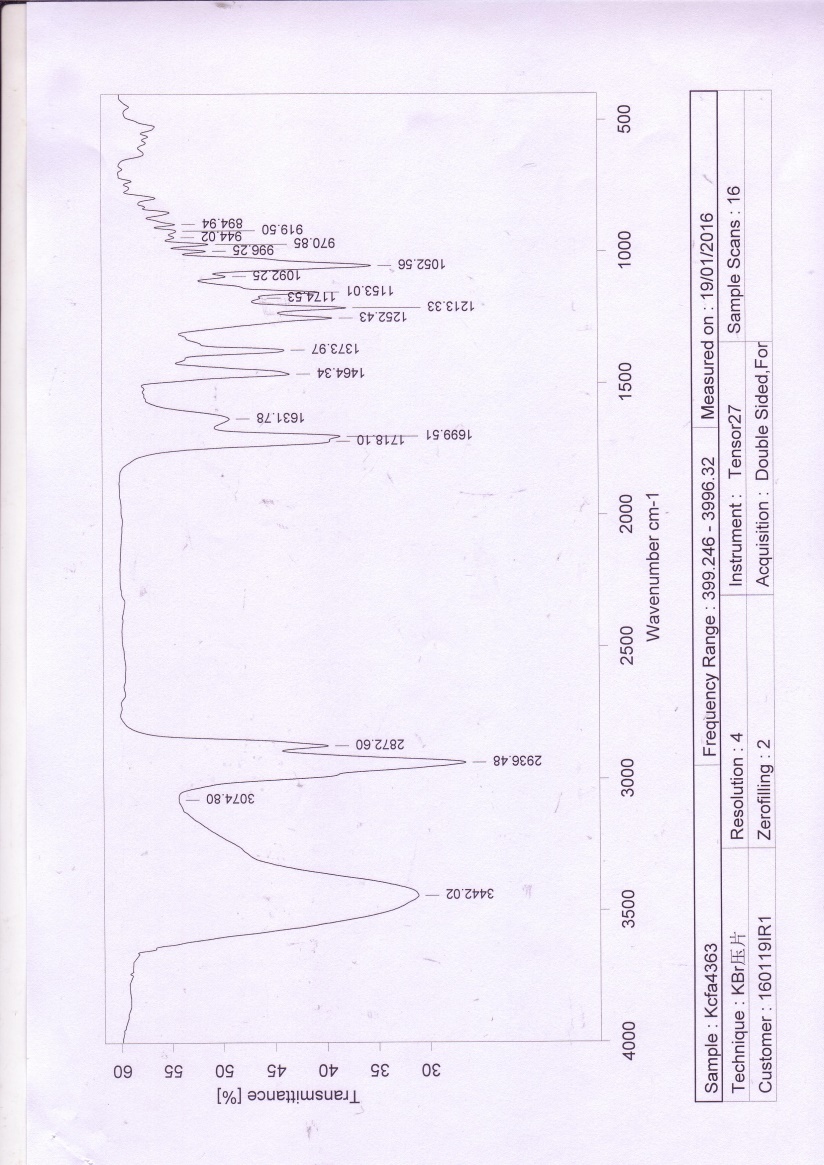

Supplement: Supplementary file 1 — Supplementary material 1 (DOCX 3746 kb) [file 13659_2016_99_MOESM1_ESM.docx]
